# Cytogenetically visible inversions are formed by multiple molecular mechanisms

**DOI:** 10.1002/humu.24106

**Published:** 2020-10-01

**Authors:** Maria Pettersson, Christopher M. Grochowski, Josephine Wincent, Jesper Eisfeldt, Amy M. Breman, Sau W. Cheung, Ana C. V. Krepischi, Carla Rosenberg, James R. Lupski, Jesper Ottosson, Lovisa Lovmar, Jelena Gacic, Elisabeth S. Lundberg, Daniel Nilsson, Claudia M. B. Carvalho, Anna Lindstrand

**Affiliations:** ^1^ Department of Molecular Medicine and Surgery, Center for Molecular Medicine Karolinska Institutet Stockholm Sweden; ^2^ Department of Clinical Genetics Karolinska University Hospital Stockholm Sweden; ^3^ Department of Molecular and Human Genetics Baylor College of Medicine Houston Texas USA; ^4^ Science for Life Laboratory Karolinska Institutet Solna Sweden; ^5^ Department of Medical and Molecular Genetics Indiana University School of Medicine Indianapolis Indiana USA; ^6^ Department of Genetics and Evolutionary Biology, Institute of Biosciences University of São Paulo São Paulo Brazil; ^7^ Human Genome Sequencing Center Baylor College of Medicine Houston Texas USA; ^8^ Department of Pediatrics Texas Children's Hospital Houston Texas USA; ^9^ Department of Clinical Genetics Sahlgrenska University Hospital Gothenburg Sweden; ^10^ Department of Clinical Genetics Linköping University Hospital Linköping Sweden; ^11^ Pacific Northwest Research Institute Seattle Washington USA

**Keywords:** chromosomal inversions, nonallelic homologous recombination, nonhomologous end‐joining, recombinant chromosomes, replication‐based repair mechanisms, whole‐genome sequencing

## Abstract

Cytogenetically detected inversions are generally assumed to be copy number and phenotypically neutral events. While nonallelic homologous recombination is thought to play a major role, recent data suggest the involvement of other molecular mechanisms in inversion formation. Using a combination of short‐read whole‐genome sequencing (WGS), 10X Genomics Chromium WGS, droplet digital polymerase chain reaction and array comparative genomic hybridization we investigated the genomic structure of 18 large unique cytogenetically detected chromosomal inversions and achieved nucleotide resolution of at least one chromosomal inversion junction for 13/18 (72%). Surprisingly, we observed that seemingly copy number neutral inversions can be accompanied by a copy‐number gain of up to 350 kb and local genomic complexities (3/18, 17%). In the resolved inversions, the mutational signatures are consistent with nonhomologous end‐joining (8/13, 62%) or microhomology‐mediated break‐induced replication (5/13, 38%). Our study indicates that short‐read 30x coverage WGS can detect a substantial fraction of chromosomal inversions. Moreover, replication‐based mechanisms are responsible for approximately 38% of those events leading to a significant proportion of inversions that are actually accompanied by additional copy‐number variation potentially contributing to the overall phenotypic presentation of those patients.

## Graphical abstract

This study indicates that short‐read whole‐genome sequencing can detect a substantial fraction of cytogenetically visible chromosomal inversions. In addition, replication‐based mechanisms are responsible for approximately 38% of those events, leading to a significant proportion of inversions that are actually accompanied by additional copy‐number variation potentially contributing to the overall phenotypic presentation of those patients.

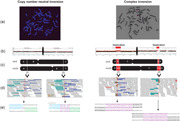

**Abbreviations**
aCGH: array comparative genomic hybridization
AF: allele frequency
BAF: B‐allele frequency
CNV: copy number variant
ddPCR: droplet digital PCR
FoSTeS: fork‐stalling and template‐switching
HI: haplotype index
IBD: identical by descent
MMBIR: microhomology‐mediated break‐induced replication
MMEJ: microhomology‐mediated end‐joining
NAHR: nonallelic homologous recombination
NHEJ: nonhomologous end‐joining
nt: nucleotide
PE: paired‐end
SNV: single nucleotide variant
WGS: whole‐genome sequencing

## 1 BACKGROUND

Inversions are a class of structural variation (SV) abundant in the human genome, first described as events involving two breakpoints and a 180° turn of the genomic segment in‐between (Kaiser, 1984). Large cytogenetically visible inversions, usually larger than 5–10 Mb, fulfill the classical definition of inversions and can be subdivided into two classes: pericentric inversions with breakpoints located on both chromosome arms, and paracentric inversions with both breakpoints on the same chromosome arm. In a clinical set, they were estimated to be as frequent as 1%–2% (de la Chapelle et al., 1974; Kaiser, 1984), with an observed *de novo* formation of 1/10,000 pregnancies (Warburton, 1991) and incidence of approximately 0.155% in an unselected newborn population (Jacobs, Browne, Gregson, Joyce, & White, 1992). Although *de novo* inversions are associated with congenital anomalies in approximately 9.6% of patients, the contribution of this particular SV in disease pathogenesis is not well understood (Warburton, 1991).

Challenges associated with the detection of large chromosomal inversions has limited our understanding of the clinical consequences for this type of structural aberration. While chromosomal karyotyping is restricted by the resolution in detecting these structural events (>5–10 Mb), next‐generation sequencing (NGS) is restrained by high rates of false‐positive and false‐negative results, requiring extensive use of orthogonal methodologies for validation (Chaisson et al., 2019; Puig, Casillas, Villatoro, & Caceres, 2015). Recent data suggest that large inversions are often flanked by genomic repeats (Chaisson et al., 2019), especially segmental duplications, contributing to both the mapping and detection challenges associated with using NGS. Smaller sized (>5‐10 Mb) (below the resolution of karyotyping but visible by molecular analysis) may also occur quite frequently (Flores et al., 2007).

**Figure 1:**
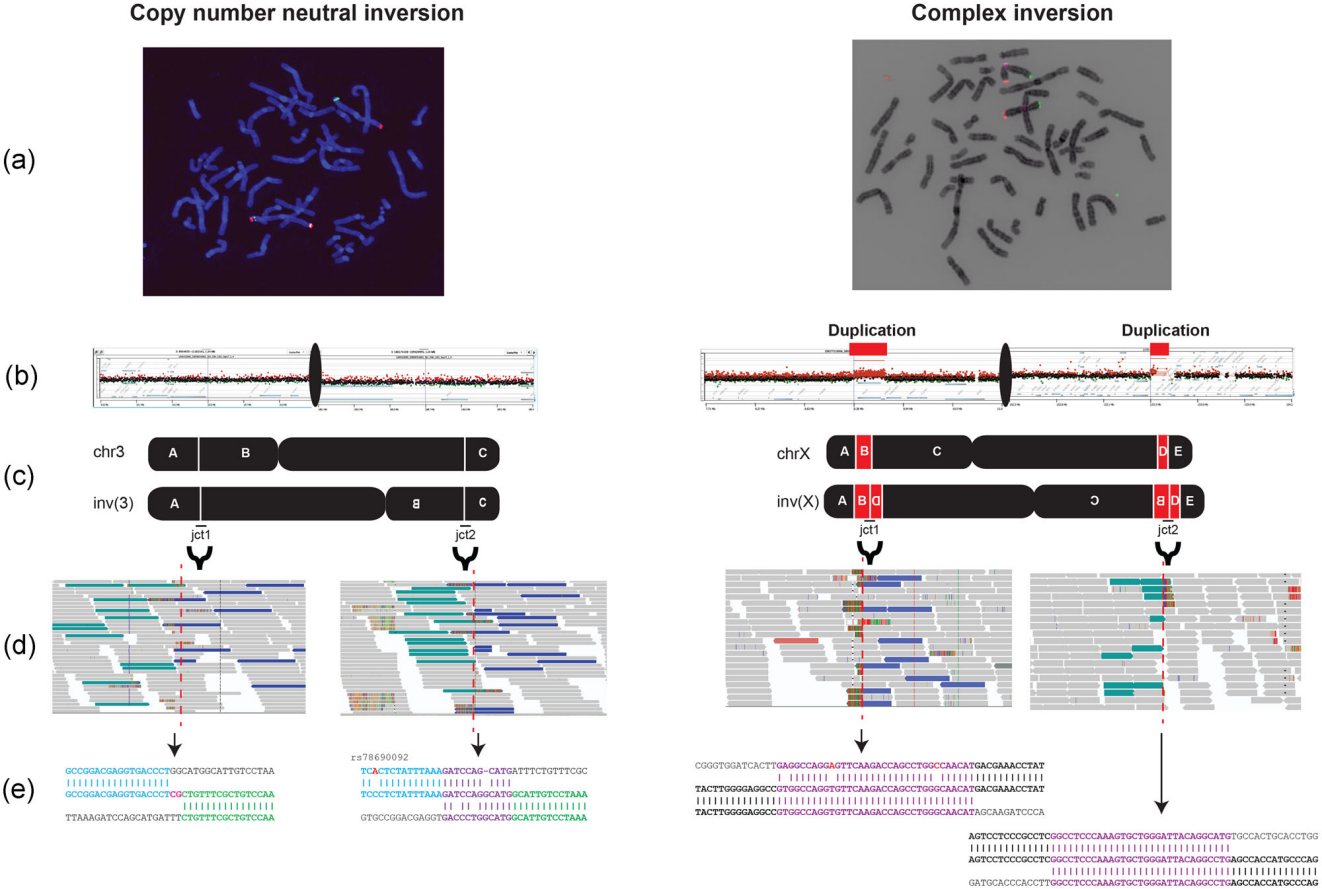
Examples of resolved classic and complex inversions using distinct methodologies. (a) Fluorescence *in situ* hybridization (FISH) data (left) showing both p and q arm probe signals in a classic heterozygous inversion case (inv(3)(p25.3q28)) initially detected by karyotyping. Two different probe colors are placed on either side of the pericentric inversion junctions allowing for confirmation of the event. In a complex inversion case (inv(X)(p22.31q28)) FISH data (right) shows the p and q probe signals switching arms. Complexities are only detected with additional experiments. (b) Array comparative genomic hybridization confirms copy number neutral state in the classic inversion case (left) but reveals the p and q arm duplications flanking the inversion in the complex case (right). (c) Proposed chromosomal architecture of the classic and complex inversion. (d) Integrative Genomics Viewer (IGV) screenshot of the classic inversion showing the discordant mapped reads as well as split‐reads clustering together. In contrast, the complex inversion does not show clustering of the discordant mapped reads as it is disrupted by a copy number event. Of note, both IGV screenshots are representative figures for such junctions in whole‐genome sequencing data. (e) Final nucleotide‐level resolution for each inversion breakpoint junction alignment based on Sanger‐sequencing for both inversion carriers.

In the cytogenetic world, inversions are classically defined as a balanced chromosomal rearrangements, that is, no gain or loss of genomic material is assumed to accompany their generation (Figure 1, left). However, smaller inversions, both unique and nonunique, forming together with kb or Mb size genomic amplifications and deletions can constitute 20%–30% of SVs in certain disease *loci*, challenging the copy‐number neutral inversion model (Beck et al., 2015; Brand et al., 2015; Carvalho et al., 2019, 2013, 2015, 2011; Figure 1, right). Supporting the observation in disease cohorts, population studies using NGS revealed that truly balanced inversions constitute a smaller fraction of the total inversions detected. Genome‐wide short‐read DNA sequencing analysis of 2504 human genomes revealed that only 20% of the validated inversions fit the definition of copy number neutral in the classical sense, that is, without gain or loss of genetic material in the breakpoints. In fact, the majority of the inversions reported therein were actually associated with copy number variants (CNVs) and classified as complex genomic rearrangements (CGRs; Sudmant et al., 2015). Recently, Chaisson et al. (2019), using a number of complementary NGS methodologies on three healthy trios, reported that approximately 25% of inversions are found embedded with CNVs, mostly copy number gains, supporting the aforementioned studies. As the included data sets from the Chaisson et al. study and the Sudmant et al. study excluded severe pediatric disease in the sequenced individuals, one could probably assume that the reported inversions constitute normal variation and can be potentially classified as benign variants (Chaisson et al., 2019; Sudmant et al., 2015). The major mechanism of formation for copy number neutral inversions has previously been proposed to be nonallelic homologous recombination (NAHR) between inverted repeats, on which large blocks of sequence homology have been estimated to explain approximately 67% of inversions (Flores et al., 2007; Kidd et al., 2008), but the formation of CGRs in a concomitant fashion suggests that other mechanisms may also play a role in their formation.

Here we investigate the genome architecture of cytogenetically detected pericentric and paracentric inversions, classically defined as copy‐number neutral, in 27 individuals. Our goals were (i) to resolve the genomic architecture of a group of large and rare “neutral” inversions by NGS and estimate the subsequent rate of associated CGRs; (ii) to establish the relative contribution of distinct molecular mechanisms underlying those large inversions; (iii) to compare the data obtained in this cohort to that of known population and disease studies to gain insights into the molecular architecture of inversions within this distinct cohort. We utilized a wide range of genomic analysis techniques including short‐read whole‐genome sequencing (WGS), linked‐read WGS, array comparative genomic hybridization (aCGH), droplet digital PCR (ddPCR), and Sanger sequencing to comprehensively characterize each case. The present study shows that high‐coverage short‐read WGS can detect a substantial fraction of cytogenetically visible inversions and resolve the majority of the breakpoints at nucleotide (nt) level resolution. In line with recent population studies, we observed that approximately 17% of apparently copy number neutral inversions are actually constituted by CGRs. The data here also indicate that, in a group of known large inversions, mechanisms distinct from ectopic recombination are relevant contributors to the formation of the majority of those events. In summary, through fully characterizing a subset of large chromosomal inversions detected through traditional cytogenetics we can more precisely define inversions at the molecular level as well as assess the underlying molecular mechanisms leading to the genesis of these chromosomal events.

## 2 METHODS

A flow‐chart detailing when each method was applied to resolve the final genomic structure of cytogenetically detected inversions is available in Figure S1.

### 2.1 Study subjects

The study cohort in total consisted of 34 individuals from 23 families, carrying 18 cytogenetically identified unique inversions (pericentric, *n* = 15, paracentric, *n* = 3) and five recombinant chromosomes (DEL/DUP) resulting from carrier mothers of heterozygous pericentric inversions. The recruitment strategy for the present study was to collect carriers of cytogenetically visible inversions where clinical data was sufficient and where genomic DNA from the patient was available. The presence or absence of a clinical phenotype was not part of the recruitment criteria, only inversion carrier status. The original mode of ascertainment and the subsequent discovery of inversion is detailed for each patient in Table 1 with karyotyping information for all 23 enrolled families. Unexpectedly, two inversions were identified in multiple unrelated individuals: inv(12)(p11.2q13), which were inherited in all cases, and inv(10)(p11.2q21), which was confirmed to be inherited in 2/5 carriers and found to likely be a rare founder variant (Gilling et al., 2006). For the remaining 16 unique inversions, 6 were confirmed to be inherited, whereas for 10 we did not have inheritance information. The recombinant chromosomes (*n* = 5) were all found to be formed *de novo* through ectopic meiotic crossing‐over in a heterozygous carrier mother.

**Table 1:** Karyotypes and mode of ascertainment of included cases

Case	Karyotype	Inversion size (% total chromosome size)	Ascertainment	Inheritance	Phenotype summary	
Pericentric inversions + generated recombinants						
P4855_207	46, XY, inv(1)(p13q25)	71.7 Mb (28.8%)	Affected phenotype	Paternal		NDD
BAB12196	46, XX, inv(3)(p25.3q28)^a^	178 Mb (90%)	Sibling of BAB12195	Maternal		Healthy
BAB12195	46, XY, rec(3)(pter→q28::p25.3→pter)mat	N/A	Affected phenotype	*De novo* recombinant		Global developmental delay, hypotonia, microcephaly, agenesis of corpus callosum, decreased global brain myelination, facial dysmorphisms, epilepsy, ONH
P2468_115	46, XX, inv(6)(p12.1q13)	22.7 Mb (13.3%)	Amniocentesis (advanced maternal age)	N.i.		Healthy
P4855_501	46, XY, inv(6)(p12q16.3)	∼41–42 Mb (∼24%)	Affected phenotype	N.i.		NDD, hearing loss, visual impairment, anosmia, hypogonadism
P5371_208	46, XY, inv(9)(p13q22)	∼47–48 Mb (∼33%)	Recurrent miscarriages	N.i.		Healthy
P4855_105	46, XY, inv(10)(p11.2q21)	23 Mb (17%)	Affected phenotype	N.i.		FHL
P4855_211	46, XY, inv(10)(p11.2q21)	23 Mb (17%)	Affected phenotype	Maternal		NDD
P5370_115	46, XX, inv(10)(p11.2q21)	23 Mb (17%)	Recurrent miscarriages	N.i.		NDD
P5370_103	46, XX, inv(10)(p11.2q21)	23 Mb (17%)	Affected phenotype	Paternal		NDD
P5370_113	46, XY, inv(10)(p11.2q21)	23 Mb (17%)	Affected phenotype	N.i.		NDD
P5513_114	46, XY, inv(10)(p12q21)	37.8 Mb (27.9%)	Affected phenotype	N.i.		NDD
P4855_144	46, XX, inv(10)(p13q11.2),	25.6 Mb (18.9%)^b^	Amniocentesis (abnormal ultrasound)	Inherited Inherited		NDD
	inv(12)(p11.2q13)	15.4 Mb (11.5%)^c^				
P4855_210	46, XY, inv(12)(p11.2q13)	15.4 Mb (11.5%)	Affected phenotype	Maternal		NDD
P4855_208	46, XY, inv(11)(p11.1q12)	∼14–15 Mb (13%)	Affected phenotype	Maternal		NDD, brother of P5370_102
P5370_102	46, XY, inv(11)(p11.1q12)	∼14–15 Mb (13%)	Affected phenotype	Maternal		NDD, brother of P4855_208
P1426_108	46, XY, inv(12)(p11.2q13)	15.4 Mb (11.5%)	Affected phenotype	Paternal		NDD
P4855_209	46, XY, inv(12)(p11.2q13)	15.4 Mb (11.5%)	Affected phenotype	Paternal		NDD
P5371_206	46, XX, inv(12)(p11.2q24.1)	69.9 Mb (52.2%)	Affected phenotype	N.i.		Cushing‐like features
P5370_201	46, XY, inv(18)(p11.3q11.2)	∼16–17 Mb (∼21%)	Affected phenotype	N.i.		Diabetes type II, Hodgkins lymphoma, hearing loss, hypogonadism, retinitis pigmentosa, acanthosis nigricans, beta thalassemia
P11758_101 (I:2)	46,X, inv(X)(p22.31q28)	144 Mb (93%)	Family investigation	N.i.		Healthy
II:1	46,X, rec(X)(pter→q28:: p22.31→pter)mat	N/A	Affected phenotype	*De novo* recombinant		Short stature (−2.5 SD), madelung deformity, short forearms and shanks, joint and skeletal pain, autism
III:3	46,Y, rec(X)(pter→q28:: p22.31→pter)mat	N/A	Affected phenotype	*De novo* recombinant		IUFD, hypoplastic and dysplastic right kidney, hydrocephalus, low‐set ears, large beaked nose
Mother of BAB3037	46,X, inv(X)(p22.2q26)	136 Mb (87%)	Child with congenital malformations	N.i.		Healthy
BAB3037	46,Y, rec(X)(pter→q26::p22.2→pter)mat	N/A	Affected phenotype	*De novo* recombinant		Tachypnea, abnormal platelet count, rhizomelic shortening, dysmorphic facial features, pectus excavatum, transverse palmar crease, hypogenitalism
Mother of BAB3038	46,X, inv(X)(p22.3q28)	142 Mb (92%)	Child with congenital malformations	N.i.		Healthy
BAB3038	46,Y, rec(X)(pter→q28::p22.3→pter)mat	N/A	Affected phenotype	*De novo* recombinant		Hypotonia, dysmorphic facial features, small hands and feet, transverse palmar creases, hypogenitalism
Paracentric inversions						
P5371_207	46, XX, inv(12)(p12.2p13.3)	15.7 Mb (11.7%)	Amniocentesis (abnormal CUB test)	Maternal	N.i. (prenatal sample), carrier mother reported healthy	
P5513_204	46, XX, inv(1)(q21.3q42.13)	75 Mb (30.1%)	Child with congenital malformations	N.i.	Healthy	
P4855_106	46, XY, inv(10)(p12.2p13.3)	∼8–9 Mb (∼6%–7%)	Family investigation	Paternal	Healthy	

Abbreviations: CUB, combined ultrasound and biochemical screening; FHL, familial hemophagocytic lymphohistiocytosis; IUFD, intrauterine fetal death; N/A, not applicable; NDD; neurodevelopmental disorder; N.i., no information; ONH, optic nerve hypoplasia.

^a^ Inversion not visible on chromosome analysis, nomenclature determined by junction sequencing.

^b^ inv(10).

^c^ inv(12).

Eighteen of the total 23 families were enrolled at the Karolinska University Hospital, Stockholm, Sahlgrenska University Hospital, Gothenburg, or Linköping University Hospital, Linköping, Sweden (Ethical Permit KS 2012/222‐31/3). One inversion carrier and one recombinant chromosome (DEL/DUP) carrier from the same family were enrolled at the University of São Paulo, São Paulo, Brazil (Ethical Permit 2589398). The present study also includes two previously published patients with recombinant chromosomes due to ectopic recombination in carrier mothers of pericentric inversions on chromosome X (Breman et al., 2011), both of whom had been referred for clinical diagnostic testing at Baylor College of Medicine, Houston, TX, USA.

In summary, study ascertainment for all families was for inversion or recombinant chromosome carrier status only. Clinical ascertainment for genetic analysis was a neurodevelopmental disorder or clinical suspicion of a syndrome concerning at least one family member in 17/23 (74%) families, 4/23 (17%) were referred due to fertility problems or prenatal testing, one (1/23, 4%) for a hematological disorder and one (1/23, 4%) for family segregation studies with a clinically affected relative.

### 2.2 Karyotyping

Metaphase slides were prepared from peripheral blood cultures according to standard protocols. Subsequent chromosome analysis was performed after G‐banding with an approximate resolution of 550 bands per haploid genome. A minimum of 10 metaphases were analyzed for each individual.

### 2.3 Short‐read WGS

Short‐read WGS was performed using Illumina 30X polymerase chain reaction (PCR)‐free paired‐end (PE; Nilsson et al., 2017) at the National Genomics Infrastructure (NGI), in Stockholm, Sweden. All data obtained were processed using NGI‐piper and analysis for structural variants was performed using the FindSV pipeline (https://github.com/J35P312/FindSV). FindSV combines CNVnator V.0.3.2 (Abyzov, Urban, Snyder, & Gerstein, 2011) and TIDDIT V.1.1.4 (Eisfeldt, Vezzi, Olason, Nilsson, & Lindstrand, 2017) and produces a single variant calling format (VCF) file, subsequently annotated by variant effect predictor (VEP) and filtered based on the VCF file quality flag (McLaren et al., 2010). Lastly, the VCF file is sorted based on a local structural variant frequency database consisting of 351 personal genome samples, and the SV of interest was identified based on the VEP annotation and variant frequency. Manual inspection and identification of split reads were performed using the Integrative Genomics Viewer (IGV; http://software.broadinstitute.org/software/igv/; Robinson et al., 2011). The exact position of breakpoints on the nt level could then be determined by alignment of split reads to the Hg19/GRCh37 reference genome using the BLAST‐like alignment tool (BLAT; https://genome.ucsc.edu/cgi-bin/hgBlat; Kent, 2002). Single nucleotide variants (SNVs) were called using the PileupPipe (https://github.com/J35P312/PileupPipe), a pipeline to perform variant calling using Freebayes (Garrison & Marth, 2012) and bcftools (Li et al., 2009), and annotation using VEP (McLaren et al., 2016). SNVs overlapping the inversions were extracted using Tabix (Li, 2011).

### 2.4 Linked‐read WGS

Linked‐read WGS was performed on seven samples (P11758_101, P4855_208, P5370_102, P4855_501, P5370_201, P5371_208, and P4855_106) using 10X Genomics Chromium at NGI. One sample (P11758_101) was sequenced for follow‐up studies, and the remaining samples were sequenced because the inversions could not be detected with short‐read WGS. Libraries were prepared using the 10X Chromium controller and sequenced on an Illumina Hiseq Xten platform as described previously (Eisfeldt et al., 2019). Data were analyzed using the default Long Ranger pipeline (https://support.10xgenomics.com/genome-exome/software/downloads/latest).

### 2.5 PCR‐specific inversion breakpoint junctions and Sanger sequencing

We designed primers to confirm the inversion breakpoint junctions (jct1 and jct2; Figure 1) obtained from the split read information derived from the WGS data from the 15 unique inversions. PCR was performed according to standard protocols using Phusion High‐Fidelity DNA Polymerase (Thermo Fisher Scientific). Each PCR was set up in pairs, one using pooled control genomic DNA (Promega) and one using the patient genomic DNA, to ensure specificity of the obtained amplicon. The same primers used for the PCR were subsequently used for Sanger sequencing each of the amplicon. Sequences were aligned using the BLAT tool (Kent, 2002) and visualized using CodonCode Aligner (CodonCode Corp). A subsequent series of primers were designed for Sanger sequencing confirmation of breakpoint junctions. Primer sequences are available in Table S1. Microhomology was considered for each junction that contained 100% nt identity between both reference strands (5′ and 3′) at the breakpoint. Microhomeology was classified for breakpoint junctions that had a shared nt similarity between 70% and 100% involving ≥5 nts with a maximum of two nt gaps (Bahrambeigi et al., 2019).

**Figure 2:**
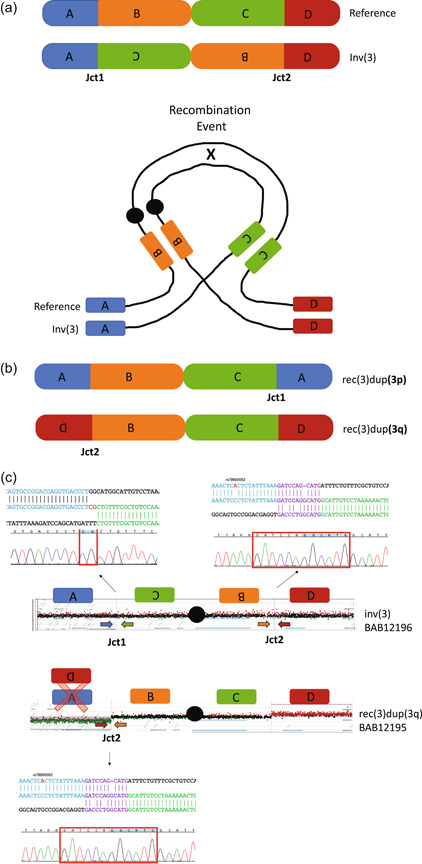
Recombinant chromosomes allow for the characterization of breakpoints in inversion carriers. (a) Reference structure as well as the inverted structure of chromosome 3 highlighting the two junctions (jct1 and jct2) with genomic segments aligned during recombination event. (b) The two possible results, rec(3)dup(3p) or rec(3)dup(3q) of a recombination event. Each result can only carry one of the junctions (either jct1 or jct2). (c) For classic inversions, where the array shows no apparent genomic alteration, we can infer the presence of both inversion junctions through mapping the location of the DEL/DUP recombinant structure. Color matching arrows representing the primer locations for each predicted junction are displayed. Using these predicted locations we were able to Sanger validate the breakpoints of jct1 and jct2 in the inversion carrier (BAB12196) as well jct2 in the recombinant chromosome (BAB12195)

For probands carrying recombinant chromosomes (DEL/DUP), we designed custom microarrays targeting chromosomes X and 3, respectively, to resolve the formation of these structures at nt level resolution. While classic inversions carry two breakpoint junctions (Figure 2a), recombinant chromosomes are predicted to carry only one out of two inversion breakpoints (jct1 or jct2; Figure 2b). We used this prediction as an approach to confirm breakpoint junctions obtained by WGS or to obtain the junctions of the recombinant chromosome whose sample was not submitted to WGS (BAB12195). To obtain jct2, outward‐facing primers were designed based on the genomic coordinates of the custom array probes mapping to the copy number neutral region upstream of the p‐arm deletion and the most centromeric probe mapping to the copy number duplication on the q‐arm (Figure 2c). Both breakpoint junctions, jct1 and jct2, were investigated in the unaffected inversion carrier sister (BAB12196). To obtain jct1 we designed an outward‐facing primer mapping to the most centromeric probe within the deleted region in the p‐arm and an outward‐facing primer at the more telomeric position within the copy number neutral region in the q‐arm (Figure 2c).

### 2.6 Array comparative genomic hybridization

A custom 2 × 400 K Agilent high‐resolution oligonucleotide microarray (AMADID: 085772) targeting the long and short arm of chromosome X was designed using the Agilent e‐array website (http://earray.chem.agilent.com/earray/; Santa Clara) to further characterize the genomic disruptions found in the family carrying an inversion and recombinant of the X chromosome. A second custom Agilent high‐resolution oligonucleotide microarray (AMADID: 085903) with a 4 × 180 K probe design targeting both arms of chromosome 3 with an average probe spacing of 1000 bp was used to characterize the family carrying an inversion and recombinant of chromosome 3. Lastly, an Agilent‐designed 1 million probe whole‐genome oligonucleotide microarray (AMADID: 021529) was performed on sample P5371_206 to confirm the CNVs detected by WGS and to rule out the presence of other potential genomic complexities.

Array experiments were performed according to the manufacturer's protocol for probe labeling and hybridization with minor modifications (Carvalho et al., 2009).

### 2.7 Droplet digital PCR

In two of the studied inversions, the copy number state of identified junctions were assayed using ddPCR. In sample P5371_206, primers were designed to specifically amplify each of the identified junctions (jct1, jct2, and jct3) to assess the relative level of each junction and in the family containing an inversion and recombinant of chromosome X, primers were designed to amplify jct2 to assess its levels across each member of the family.

Both assays were performed using a QX200 AutoDG ddPCR System from Bio‐Rad following normal protocols for an EvaGreen reaction. A final volume of 21 μl was generated for each PCR reaction using 10 μl Q200 EvaGreen Supermix, 0.5 μl of both the forward and reverse primer (10 μM) as well as 30 ng of genomic DNA. The reaction mix was briefly subjected to centrifugation before droplet generation was performed on the Bio‐Rad QX200 AutoDG. Droplets were transferred to a standard thermocycler and the PCR performed using the following cycling conditions with a 2°C per second ramp rate for all steps: 5 min at 95°C, 40 cycles of (30 s at 95°C, 1 min at 65°C, 1 min at 72°C), 5 min at 4°C, 5 min at 90°C, and lastly, infinite hold at 12°C. Positive droplets for each reaction were then quantified and interrupted using the QuantaSoft software suite from Bio‐Rad.

### 2.8 Haplotype analysis of founder inversion carriers

To investigate the hypothesis that carriers of the founder inversions (inv(10)(p11.2q21) and inv(12)(p11.2q13)) would share common haplotypes, we used WGS data from the carriers to identify SNVs for haplotype analysis. For the inversion on chromosome 12, four individuals were analyzed: P1426_108, P4855_144, P4855_210, and P4855_209, two of them related (P4855_144 is the mother of P4855_210) whereas, for chromosome 10, five individuals were analyzed: P4855_105, P4855_211, P5370_115, P5370_103, and P5370_113, all unrelated to our knowledge.

First, we generated VCF files consisting of all homozygous SNVs as well as all heterozygous SNVs with allele frequency (AF) less than 0.25 as based on the max_AF flag in VEP (McLaren et al., 2016) on chromosome 10 or 12 that were present in all individuals carrying the identified inversions. The threshold of AF < 0.25 was chosen because the probability of all individuals carrying the same SNV by chance would be 0.25^3^ (*p* = .016) (inv(12)) or 0.25^5^ (*p* = .001) (inv(10)), respectively.

Next, the similarity of SNV overlapping the inversions in the unrelated carriers was calculated and compiled into heatmaps. This analysis was performed using hierarchical clustering, using the heatmap2 package of GGplot (Wickham, 2016). The clustering was based on the haplotype index (HI), a metric similar to the Jaccard index (Appendix S1). The HI was calculated for each pairwise combination of individuals, producing a similarity matrix of the same size as the number of individuals. The clustering was performed using the resulting matrix as input, and the Pearson correlation between individuals was used as a distance metric.

The haplotypes of the inv(12) and inv(10) carriers were analyzed separately. Hence, the analysis was performed twice and compared to the same control individuals.

The significance of the clusters was tested using the Mann–Whitney *U* test.

### 2.9 Phasing inversion and flanking duplications

The duplications flanking the large pericentric inversion in the inv(X) carriers were phased using 10X Genomics Chromium linked‐read WGS data. The B‐allele frequencies (BAFs) of heterozygous SNVs within the duplication were correlated with SNVs found within molecules spanning the inversion breakpoints.

Briefly, for SNVs within the duplication, frequency will depend on whether the SNVs are present on the duplicated or nonduplicated copy. Hence, BAF will be either approximately 66% (present in two out of three copies) or 33% (present on one out of three copies). This information can then be used to determine whether the inversion is in *cis* with either of the duplications and if so, all informative SNVs from the molecules spanning the inversion breakpoints will have a BAF of approximately 66% of reads. Conversely, the duplications and the inversion are assumed to originate from different alleles if the informative SNVs on such molecules are present in approximately 33% of reads. Phased molecules and informative SNVs were identified by manual inspection of barcodes and nt changes in the IGV browser.

## 3 RESULTS

### 3.1 Short‐read WGS can identify majority of the breakpoint junctions for large inversions

A total of 18 unique inversions, previously detected by karyotyping, were included in the cohort of the present study. Out of the total, 11 pericentric and two paracentric (13/18, 72%) had at least one junction resolved to the nt level whereas 11/18 (61%) had both junctions resolved (Table 2).

**Table 2:** Breakpoint junction location, features, and inferred mechanism of formation

Sample ID	Karyotype	Junction 1	Features Junction 1	Junction 2	Features junction 2	Additional junctions/SVs	Mechanism Jct1/Jct2
Pericentric inversions							
P4855_144	46, XX, inv(10)(p13q11.2)	chr10:17514291 (Intergenic)	Chr10p: 0 bp DelInv10pq: 3 bp MicrohomologyChr10q: 0 bp Del	chr10:17514287(Intergenic)	Chr10p: 0 bp DelInv10pq:13 bp Imperfect Templated InsChr10q: 0 bp Del	No	MMEJ/MMBIR
		chr10:43162134(L1PA4)					
P4855_144,P1426_108, P4855_210, P4855_209	46, XX/XY, inv(12)(p11.2q13)	chr12:32819401(*AluSx3*)	Chr12p: 0 bp DelInv12pq: Blunt Chr12q: 0 bp Del	chr12:32819402(*AluSx3*)	Chr12p: 0 bp DelInv12pq: 1 bp MicrohomologyChr12q: 0 bp Del	No	NHEJ/MMEJ
		chr12:48237160(3UTR VDR)		chr12:48237156(3UTR VDR)			
P4855_211, P5370_115, P5370_103,P5370_113, P4855_105	46, XX/XY, inv(10)(p11.2q21)	chr10:37108082(Intergenic)	Chr10p: 0 bp DelInv10pq: 3 bp MicrohomologyChr10q: 0 bp Del	chr10:37108082(Intergenic)	Chr10p: 0 bp DelInv10pq: Blunt Chr10q: 0 bp Del	No	NHEJ/MMEJ
		chr10:60078188(Intergenic)		chr10:60078189(Intergenic)			
P11758_101	46,X, inv(X)(p22.31q28)	chrX:9388053 (*AluJr*)	ChrXp: 0 bp DelInvXpq: 28 bp MicrohomologyChrXq: 0 bp Del	chrX:9736949(*AluSz6*)	ChrXp: 0 bp DelInvXpq: 32 bp MicrohomologyChrXq: 0 bp Del	350 kb Xp22.31p22.2(9388054–9737230)x3 58 kb Xq28(153378509–153436856)x3	*Alu‐Alu* mediatedComplexMMBIR/*Alu‐Alu* mediatedComplexMMBIR
		chrX:153378508 (*AluSx1*)		chrX:153436875(*AluJo*)			
P5371_206	46, XX, inv(12)(p11.2q24.1)	chr12:27910978(Simple repeat)	Chr12p: 5 bp DelInv12pq: Blunt Chr12q: 0 bp Del	chr12:27918993(Intron MANSC4)	Chr12q: 0 bp DelInv12pq: 2 bp InsChr12p: 0 bp Del	Jct3:chr12:27910984chr12:97848053(*AluJo*) – Chr12p: 5 bp DelInv12pq: 4 bp microhomologyChr12q: 0 bp Del7.9 kb12p11.22(27910906–27918929)x33.8 kb 12q23.1(97844238–97848048)x125 kb12q23.1(97847893–97873452)x3	ComplexMMBIR/ComplexMMBIR/ComplexMMBIR
		chr12:97844244(L1MA4)		chr12:97873391(*AluJr*)			
P2468_115	46, XX, inv(6)(p11q13)	chr6:5298058(Intergenic)	Chr6p: 2 bp DelInv6pq: 6 bp microhomologyChr6q: 15 bp Del	chr6:52981061(Intergenic)	Chr6p: 2 bp DelInv6pq: 3 bp MicrohomologyChr6q: 15 bp Del	No	MMEJ/MMEJ
		chr6:75693677(Intergenic)		chr6:75693693(Intergenic)			
P5513_114	46, XY, inv(10)(p12q21)	chr10:22020626(Intron *MLLT10*)	Chr10p: 3 bp DelInv10pq: 1 bp InsChr10q: 0 bp Del	chr10:22020630(Intron *MLLT10*)	Chr10p: 3 bp DelInv10pq: Blunt Chr10q: 0 bp Del	No	NHEJ/MMEJ
		chr10:59866350(Intergenic)		chr10:59866351(Intergenic)			
P4855_207	46, XY, inv(1)(p13q25)	chr1:113466005(Intron *SLC16A1*)	Chr1p: 0 bp DelInv1pq: 2 bp MicrohomologyChr1q: 0 bp Del	chr1:113466004(Intron *SLC16A1*)	Chr1p: 0 bp DelInv1pq: 2 bp MicrohomologyChr1q: 0 bp Del	No	MMEJ/MMEJ
		chr1:185145627(Intron *SWT1* (L2b))		chr1:185145626(Intron *SWT1* (L2b))			
BAB12196	46, XX, inv(3)(p25.3q28)	chr3:10558064(Intergenic)	Chr3p: 0 bp DelChr3pq: 2 bp InsChr3q: 0 bp Del	chr3:188797978(ERVL)	Chr3p: 0 bp DelInv3pq: 12 bp MicrohomologyChr3q: 0 bp Del	No	MMEJ/MMEJ
		chr3:188797973(ERVL)		chr3:10558065(Intergenic)			
Mother of BAB3037^a^	46,X, inv(X)(p22.2q26)	N/A	N/A	ChrX:5671604(Intergenic)	ChrXp: 0 bp DelChrXpq: 9 bp + 59 bp Templated InsChrXq: 0 bp Del	No	‐‐‐‐/MMBIR
				ChrX:141567047(Intergenic)			
Mother of BAB3038^a^	46,X, inv(X)(p22.3q28)	N/A	N/A	ChrX:6435909(Intergenic)	ChrXp: 0 bp DelInvXpq: 8 bp Templated InsChrXq: 0 bp Del	No	‐‐‐‐‐/MMBIR
				ChrX:149207269(Intergenic)			
Paracentric inversions							
P5371_207	46, XX, inv(12)(p12.2p13.3)	chr12:6338819(Intron *CD9*)	Chr12p: 4 bp DelInv12pp: Blunt Inv12p: 1 bp Del	chr12:6338824(Intron *CD9*)	Chr12p: 4 bp DelInv12pp: Blunt Chr12p: 1 bp Del	No	NHEJ/NHEJ
		chr12:22046497(intron of *ABCC9*/L1MEA4)		chr12:22046499(Intron of *ABCC9*/L1MEA4)			
P5513_204	46, XX, inv(1)(q21.3q42.13)	chr1:154623692(MLT1A1/ERVL‐MaLR)	Chr1q: 527 bp DelInv1qq: 1 bp MicrohomologyChr1q: 10 bp Del	chr1:154624219(MLT1A1/ERVL‐MaLR)	Chr1q: 527 bp DelInv1qq: 1 bp MicrohomologyChr1q: 10 bp Del	527 bp Del	MMEJ/MMEJ
		chr1:229644659(L2c)		chr1:229644649(L2c)			

*Note*: Nucleotide resolution coordinates are in Hg19

SVs were considered when larger than 100 bp in size.

Abbreviations: Del, deletion; Ins, insertion; MMBIR, microhomology‐mediated break‐induced replication; MMEJ, microhomology‐mediated end joining; N/A, not applicable; NHEJ, nonhomologous end joining.

^a^ Inferred junction based on recombinant chromosome in child.

Short‐read PE WGS (Nilsson et al., 2017) was performed on 15 unique inversions and three inversions were analyzed using a dual‐strategy of aCGH and breakpoint PCR/Sanger sequencing starting from the recombinant chromosome (Figure 2). Short‐read PE WGS fully resolved the breakpoint junctions in 10/15 unique inversions (67%), all junctions were supported by split reads and independently confirmed by an orthogonal experimental approach (breakpoint PCR and Sanger sequencing; Figure S2 and Table S1). Five cytogenetically visible inversions in five carriers (Table 1; P4855_208, P4855_501, P5370_201, P5371_208, and P4855_106), could not be resolved by utilizing either WGS method. The exact coordinates for the resolved breakpoint junctions are presented as molecular karyotypes in Table S2.

For the three inversions where breakpoint junctions were resolved using aCGH and Sanger sequencing (Figure 2), we obtained the inversion breakpoint junctions by inferring the relative location of the junction using the CNV information from high‐resolution custom arrays from the probands carrying the recombinant chromosomes (DEL/DUP; Figure 2). Genomic DNA from inversion carriers of the same family were used to confirm jct2 and to obtain jct1. This approach successfully resolved jct1 and jct2 in the family carrying inv(3)(p25.3q28) (Table 1; BAB12195 and BAB12196; Figures S2 and 2, Table 2).

**Figure 3:**
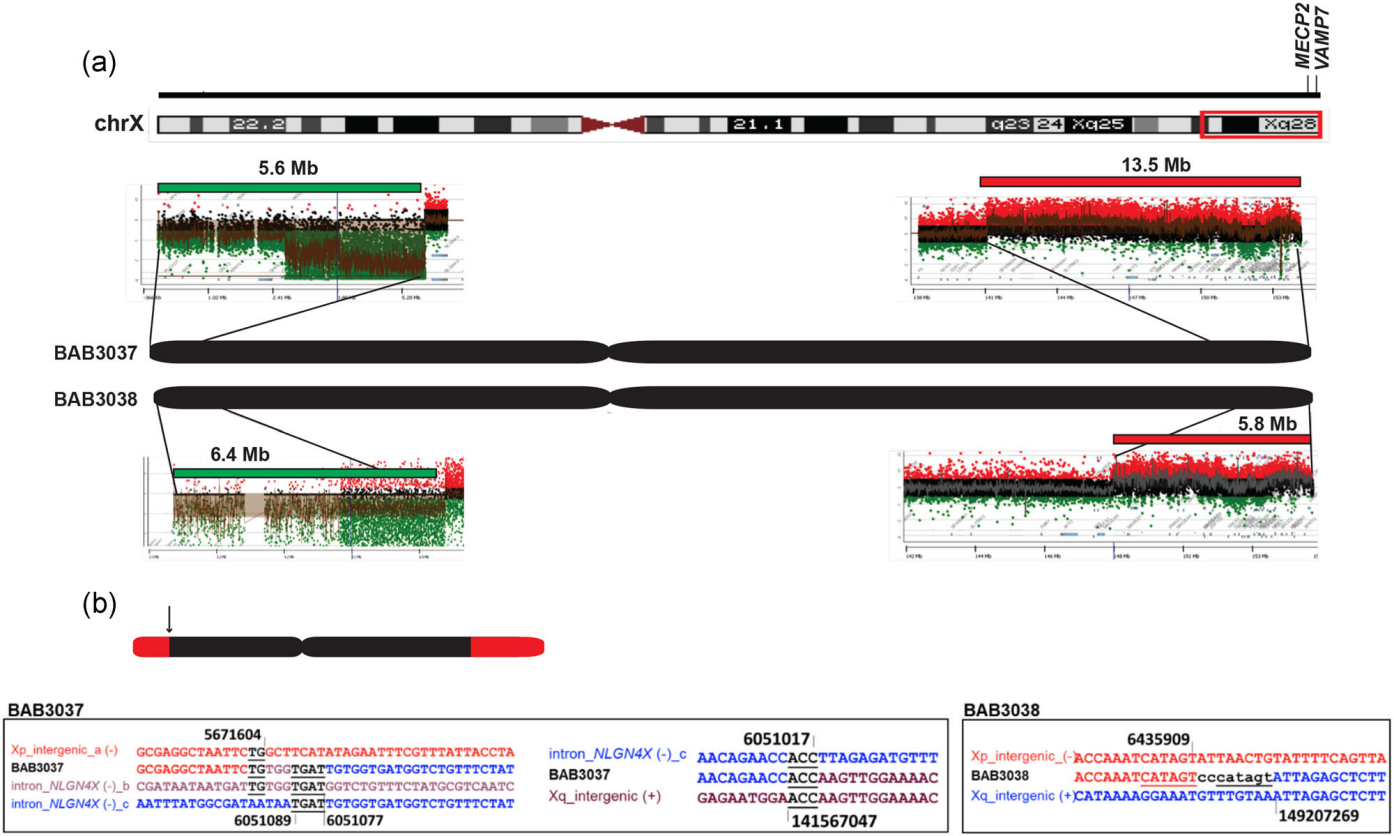
Nuclotide‐level resolution for jct2 was obtained in two individuals with a recombinant chromosome X. (a) Custom aCGH showing DEL/DUP structure of recombinant chromosome X in patients BAB3037 and BAB3038. (b) Sanger sequencing of jct2 was obtained from individual‐specific PCR products based on aCGH CNV positions. Sequencing revealed microhomology (bold black) and templated insertions (see text for details) suggesting replicative mechanism such as MMBIR underlies the formation of the origional inversions. aCGH, array comparative genomic hybridization; MMBIR, microhomology‐mediated break‐induced replication

In two families with cytogenetically detected pericentric inversions involving chromosome X, we were able to obtain only jct2 in both of the probands with X‐chromosome recombinants (BAB3037 and BAB3038; Figure 3 and Table 2; Breman et al., 2011). We did not have access to maternal DNA to confirm the presence of jct2 and to obtain the predicted jct1 in those two cases. Both BAB3037 and BAB3038 are severely affected males due to the duplicated segments on Xq that includes *MECP2*, a known intellectual disability syndrome gene (MIM# 300260; Breman et al., 2011).

At least 16 of the inversion carriers are clinically affected (no clinical information was available for P5371_207), ranging from mild (neurobehavioral conditions, mild learning difficulties) to severe (intellectual disability, developmental delay, autism; Table 1). Gene disruptions detected through precise breakpoint mapping does not substantially explain the phenotypic outcomes for these patients, however their possible positional effects were not scrutinized.

### 3.2 CNVs are formed concomitantly with apparently balanced inversions

**Figure 4:**
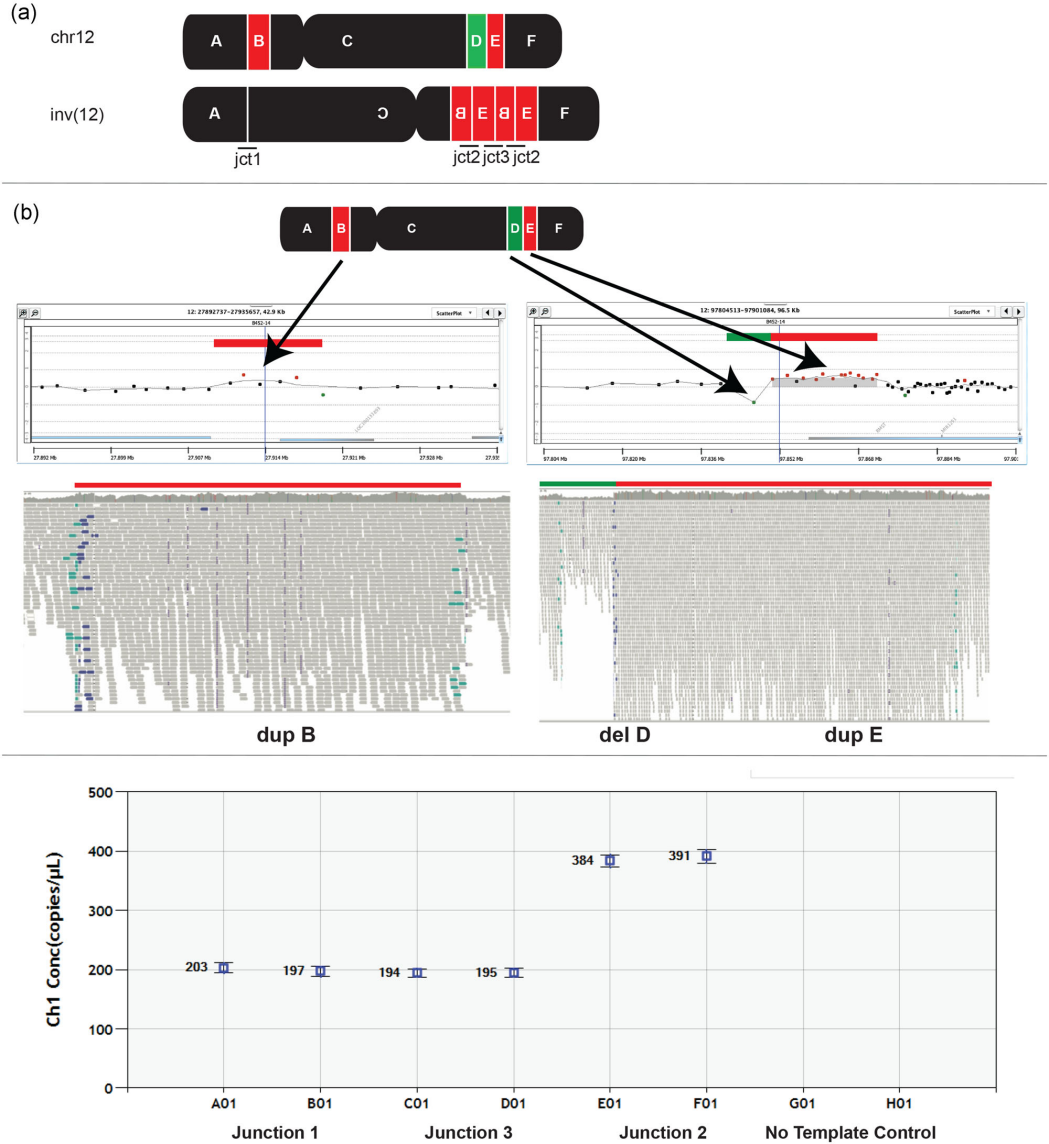
Unexpected complexity in P5371_206 revealed by whole‐genome sequencing (WGS) and array comparative genomic hybridization. (a) WGS revealed a complex rearrangement in individual P5371_206 with a pericentric inversion on chromosome 12 (inv(12)(p11.2q24.1)), which appeared to be balanced on karyotyping. The rearrangement consisted of six genomic segments, of which two were duplicated (red segments B and E) and one was lost (green segment D). (b) A 1 M microarray confirmed the duplications and the deletion that had first been identified by WGS. Screenshots from Agilent Technologies Genomic Workbench microarray software (top, B) and Integrative Genomics Viewer (below, B). (c) Droplet digital PCR confirmed the structure of the chromosome with junction 2 present twice.

Out of the total number of unique inversions, 3/18 (17%) were found to be unbalanced considering CNVs larger than 100 bp in the breakpoint junctions (Table 2). In patient P5513_204, a deletion of 527 bp was detected that may have resulted from two double‐stranded breaks in close proximity. The pericentric inversion inv(12)(p11.2q24.1) in individual P5371_206 was found to have additional CNVs at both inversion junctions (Figure 4a,b). The identified CNVs in this individual consisted of a small deletion (D: 3.8 kb) from a segment at 12q23.1 and two copy number gains consisting of duplicated segments, B: 7.9 kb at 12p11.22, and E: 25 kb at 12q23.1 at jct2, (Figure 4 and Table 2). Remarkably, jct2 (Figure 4) was amplified and inserted back at 12q23.1 which led to the deletion of the D segment and formation of a new junction (jct3). The resolved structure of this complex inversion was confirmed by ddPCR which showed jct2 at twice the levels of jct1 and jct3 (Figure 4c).

**Figure 5:**
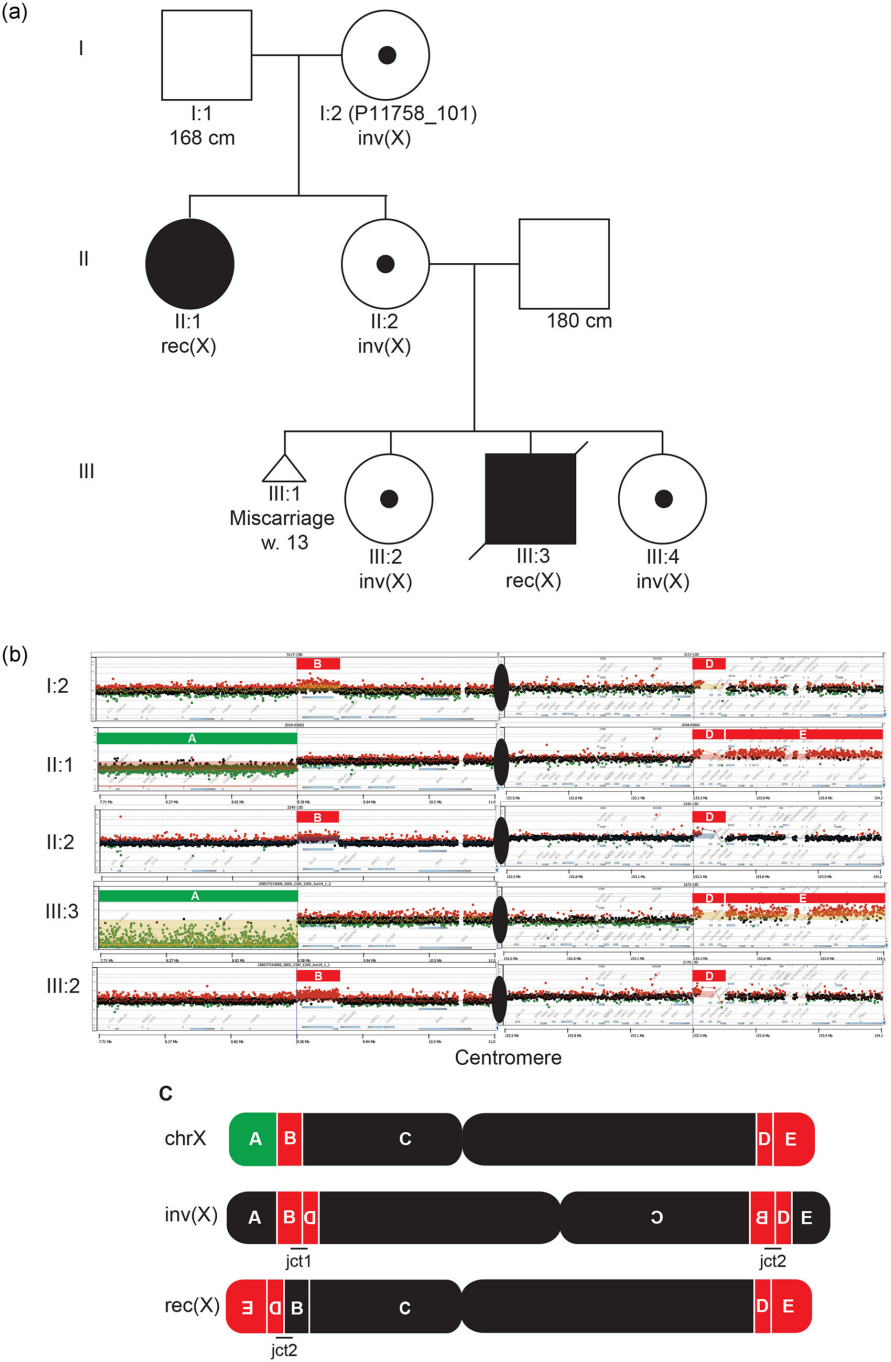
Complex pericentric inversion on chromosome X, segregates in three generations and produces two independent recombinant chromosomes. (a)The family was referred for clinical investigation due to an intrauterine fetal death in gestational week 40 (III:3), which revealed an apparently balanced inv(X)(p22.31q28) in four individuals, and an unbalanced recombinant chromosome in the fetus as well as the sister of the proband. (b) The targeted array comparative genomic hybridization (aCGH) analysis provided with detailed information on the structure of the rearranged chromosomes in both inversion and recombinant chromosome carriers in the family. The duplications were found to originate from the same allele as the inversion and had hence been formed concomitantly with the inversion. (c) The proposed genomic architecture for both the inversion and recombinant chromosome using aCGH and whole‐genome sequencing revealed additional complexity with two duplications on each side of the inversion (red segments B and D).

**Figure 6:**
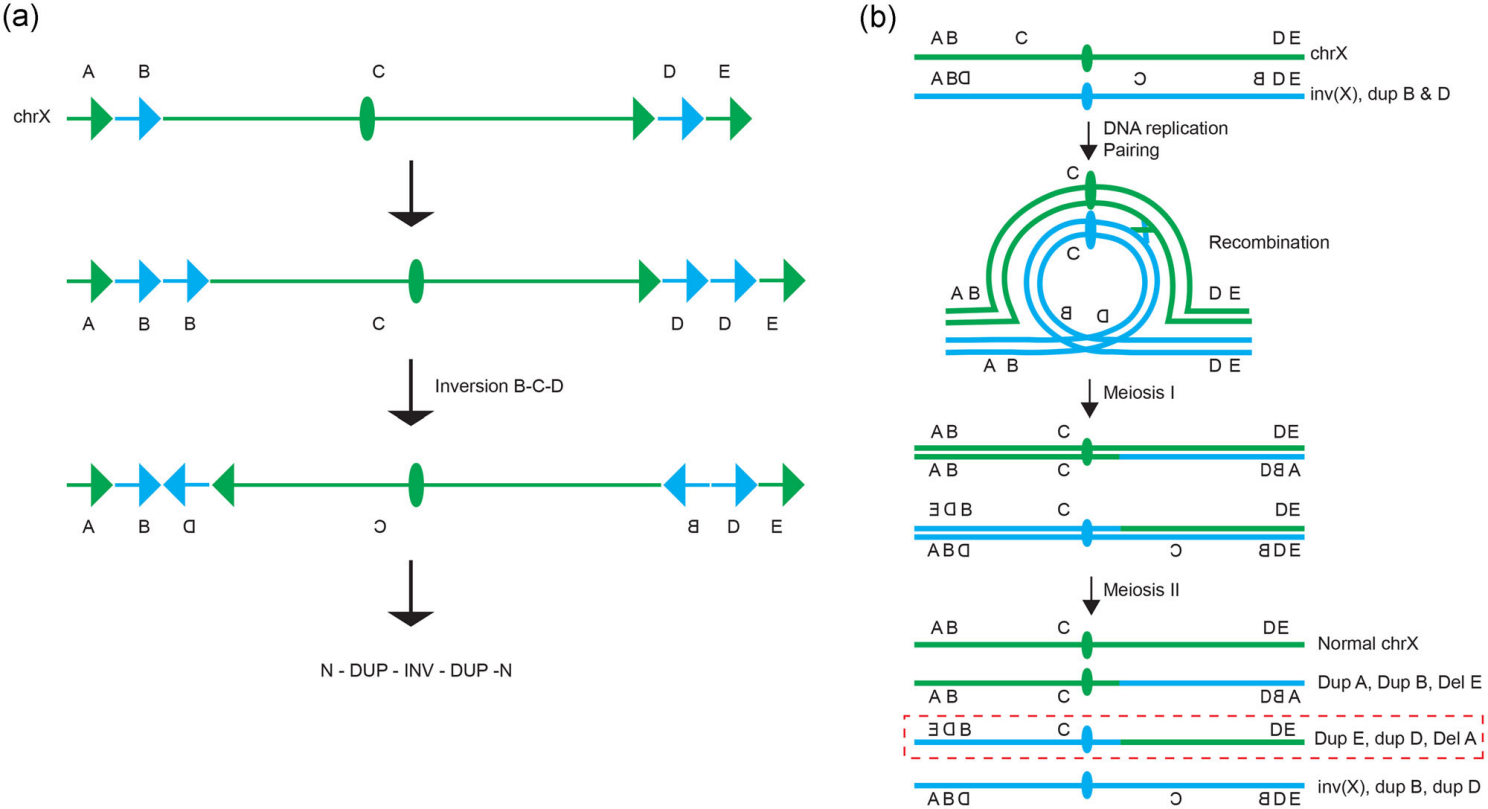
Proposed mechanism of formation of inv(X) with additional complexities and formation of unbalanced recombinants. (a) The karyotypically balanced inv(X) was found to have two duplications flanking the inversion (DUP–INV–DUP). Phasing of the duplications B and D supported the hypothesis that the duplications had formed concomitantly to the inversion. (b) The family history revealed that two individuals in the family had the same unbalanced recombinant chromosome formed through recombination between the normal allele and the allele with inversion, with duplication of segments D and E and deletion of segment A. The recombinant chromosome in this family is highlighted by the dashed red line

The second unbalanced pericentric inversion was detected in a family carrying an inv(X)(p22.31q28) that segregates in four family members over three generations (Table 1, Figures 5 and S3). This inversion independently generated two identical recombinant chromosomes, 46,X, rec(X)(pter ‐> q28::p22.31 ‐>pter)mat in generation II and III in this family (Figure 5). Inversion carriers present variable clinical phenotypes, whereas carriers of the recombinant chromosomes are severely affected (Figures 5a and S4). In‐depth characterization of the inversion structure revealed that this seemingly balanced inversion harbored additional complexities. To identify the precise breakpoints on the inverted X chromosome, we used high‐resolution aCGH to map the breakpoint regions in combination with WGS data analysis (Figure 5b,c). This combined analysis enabled resolving the genomic structure since the complexity of Xq28 locus hampered our ability to properly identify the split reads in the WGS data. The Xq28 locus includes the Opsin/*TEX28* array, a region consisting of long stretches of low‐copy repeats responsible for the majority of genomic breaks at that locus (Carvalho et al., 2009). In the inversion carriers (I:2, II:2, III:2, and III:4; Figure 5a) aCGH and WGS revealed a 350 kb duplication at the breakpoint on Xp22.31 involving two genes, *TBL1X* and *GPR143* (Segment B), and a 58 kb duplication on Xq28 involving the Opsin/*TEX28* array (Segment D). Split read analysis followed by Sanger sequencing confirmation revealed that the duplication and inversion junctions are the same, suggesting that they were formed in the same event constituting a DUP–INV–DUP structure. Analysis of molecules bridging both duplications by linked‐read sequencing showed that they were present on the same allele in *cis*. This result along with the segregation of both SVs by all carriers, support the contention that the inversion and duplications were formed together in a single event (Figure S5). Finally, the recombinant chromosomes formed recurrently in generation II and III (II:1 and III:3) are predicted to result from meiotic ectopic crossing‐over involving homologous chromosomes heterozygous for the inv(X)(p22.31q28), which generated the recombinant chromosomes twice in this family (Figure 6).

Such complex structures consisting of an inversion flanked by duplications, DUP–INV–DUP, was observed previously in a report of another pericentric inversion involving chromosome 7 and it is similar to other complex rearrangements involving paracentric inversions, termed DUP–NML–INV/DUP (Carvalho et al., 2012; Gu et al., 2015; Yuan et al., 2015). Jct2 was mediated by *Alu–Alu* recombination between an *Alu*Jr and an *Alu*Sx1 sharing 35% of nt similarity which produced an *Alu*–*Alu* fusion (Figure S2). Formation of complex inversions by *Alu–Alu* recombination was previously observed in similar paracentric complex inversions (Gu et al., 2015).

The clinical presentation of the inversion carriers in the family ranged from none (*n* = 1) to slightly disproportionate short stature with or without diffuse joint pain (*n* = 2). One balanced inversion carrier (III:4; Figure 5a) was a newborn at the time of clinical investigation and has no potentially clinically relevant phenotypes reported. We note phenotypic discrepancies in the inv(X) family that seemed to worsen over generations in the carriers of the balanced inversion (grandmother I:2 is healthy and of normal height, mother II:2 has short stature and daughter III:2 has disproportionate short stature and diffuse joint/skeletal pain; Figure S4). To investigate the possibility of mosaicism for the inversion on chromosome X in the grandmother I:2 (P11758_101), we performed ddPCR targeting jct2 but found no evidence for this hypothesis (Figure S4). All inversion carriers are females and differences in X‐inactivation would be a plausible mechanism underlying the phenotypic discrepancies in the carriers, however X‐inactivation status was not scrutinized in these patients.

In summary, duplication and deletions associated with formation of pericentric inversions were observed in three cases (inv(12)(p11.2q24.1), inv(X)(p22.31q28), and inv(1)(q21.3q42.13)). The size of the duplications varied from 59 bp to 350 kb in size, whereas the deletions varied from 527 bp to 3.8 kb in size.

### 3.3 Breakpoint junction feature implicate mechanisms of inversion formation

Out of the total breakpoint junctions where we were able to obtain nt‐level resolution (*n* = 25; Table 2), the majority of the breakpoint junctions of the cytogenetically visible inversions (17/25, 68%) showed junctional features that appeared to suggest nonhomologous end‐joining (NHEJ; *n* = 5) or microhomology‐mediated end‐joining (MMEJ; *n* = 12) as a mechanism of formation with blunt fused ends or short microhomology ranging from 1 to 6 bp, and nontemplated small insertions of random nts in five inversion junctions (Figure S2 and Table 2). One inversion (inv(6)(p11q13); P2468_115) had small deletions of 2 and 15 nts at the junctions in addition to 3 and 6 nts microhomology in the junctions, respectively, suggestive of MMEJ. In contrast, 8 out of 25 (32%) breakpoint junctions (i.e., 5 inversions out of 13, 38%) presented features consistent with MMBIR, such as concomitant generation of templated insertions (P4855_144, mother of BAB3037, mother of BAB3038) and CNVs (P11758_101) as well as *Alu–Alu* recombination (P5371_206).

We reanalyzed seven additional previously published unique inversions, also visible on karyotype, which had available sequencing data of the junctions (Chiang et al., 2012; Watson et al., 2016). In those inversions, microhomology of 2–3 nts was observed in two junctions (14%) and insertions of 1–3 random nts was observed in an additional two junctions (14%). A templated insertion was observed in one junction and a rare SNV in the proximity of the junction was observed in one case. The same case harboring the rare SNV also had a deletion in one breakpoint. Five junctions presented blunt end‐joining (Figure S6). Previously published data suggest that duplications or templated insertions at the junctions of large inversions are observed in approximately 1 out of 7 cases or approximately 14%.

Smaller duplications (<100 bp) were observed at jct2 of the recombinant chromosomes in probands BAB3037 and BAB3038. In these two cases, Sanger sequencing revealed insertions of templated segments copied from the Xp 3′ end as in BAB3038 (CCCATAGT) or from a Xp locus as far as 380 kb as observed at jct2 in BAB3037 (small insertion of TGTGGTGAT followed by an insertion of 59 bp, both segments originated from within an intron of gene *NLGN4X* [Figure 3 and Table S3]).

### 3.4 Two founder inversions, inv(10)(p11.2q21.2) inv(12)(p11.2q13), detected in multiple unrelated cases

**Figure 7:**
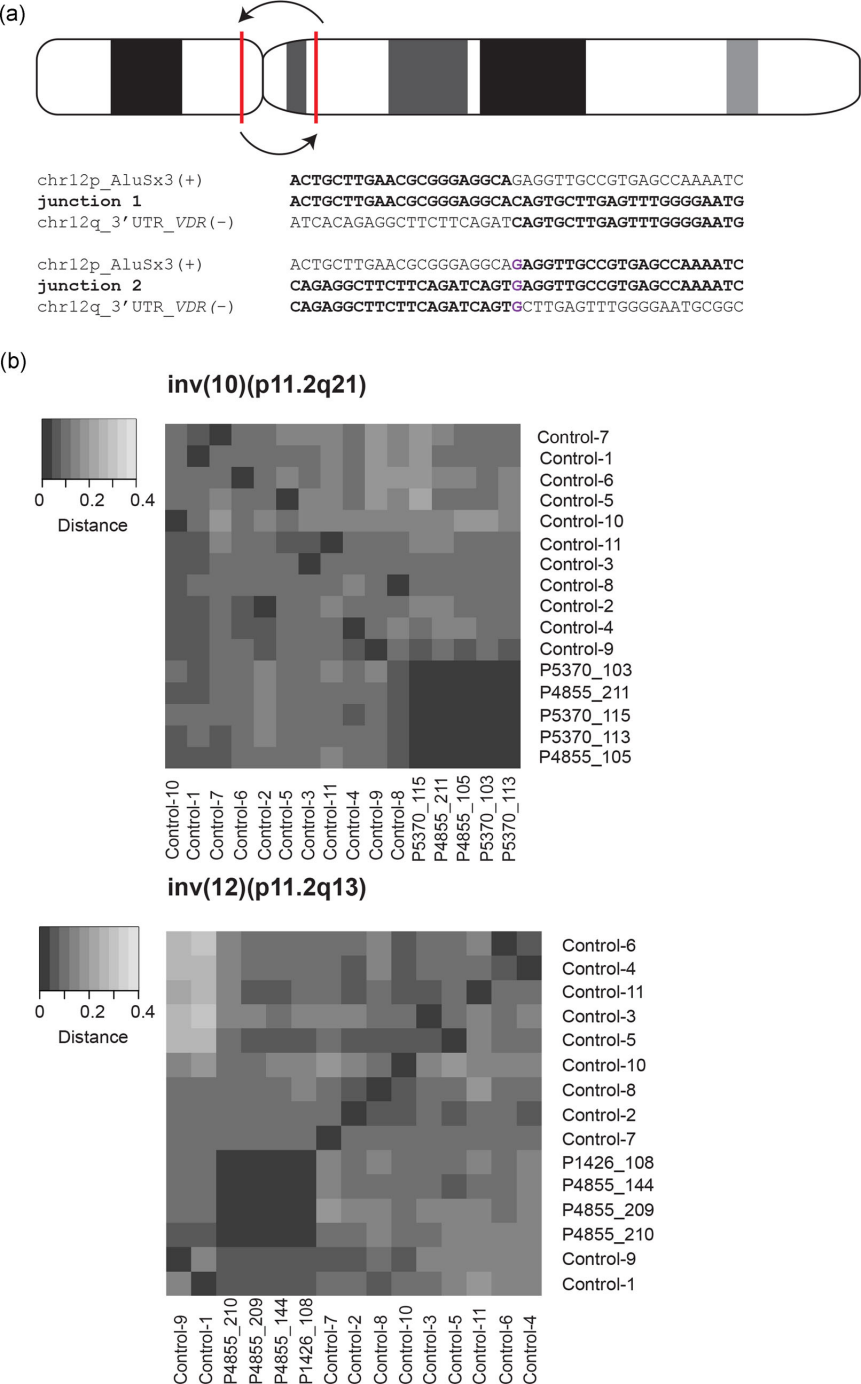
Two founder inversions detected in multiple unrelated individuals. (a) The pericentric inversion on chromosome 12, inv(12)(p11.2q13), was identified in three unrelated Swedish families with identical breakpoint junctions in all individuals. (b) In addition to the inv(12) founder inversion, a previously published and known founder inversion (Gilling et al., 2006) was identified in the cohort (inv(10)(p11.2q21)) (breakpoint junctions: Figure S7). Heatmaps were generated through analysis and comparison of haplotypes performed on all founder inversion carriers, and 11 unrelated individuals of Swedish descent. Both analyses showed that the founder inversion carriers shared a significant amount of common haplotypes and clustered tightly. Distance; the fraction of dissimilar single nucleotide variants (SNVs) between individuals. The darker color indicates a higher amount of shared SNVs

Two pericentric inversions with identical jct1 and jct2 were found in nine individuals. Inv(10)(p11.2q21.2) was detected in five unrelated individuals, whereas inv(12)(p11.2q13), was detected in four individuals, three of them are unrelated. The same inv(10) was reported previously as a founder inversion among northern Europeans (Gilling et al., 2006; Figure S7). An inv(12)(p11.2q13) was reported twice in 1986 as a possible founder variant in southern Germany (Voiculescu et al., 1986), and in three individuals of Swedish and Danish descent in another study (Sherman et al., 1986). As the breakpoint junctions of these inversions have not been characterized, we can only speculate that these inversions are the same as the inv(12)(p11.2q13) presented here. Further investigation of the founder variant hypothesis indicated that both inv(10)(p11.2q21.2) carriers and inv(12)(p11.2q13) carriers shared a significant amount of both common and more rare haplotypes, compared to 13 unrelated individuals of Swedish origin (*p* values 2.8e−07 for inv(10) and 1.8e−08 for inv(12)) (Figure 7). In the context of the present study, the founder element of these two inversions was only investigated for the purpose of excluding that the inversions occurred recurrently in unrelated individuals. On the contrary, the data clearly indicate that those inversion carriers had a common ancestor and there is no data supporting a clinical contribution of those inversions thus far.

Lastly, we examined the SweGen data set (Ameur et al., 2017), consisting of WGS data from 1000 Swedish individuals, and gnomAD‐SV (Collins et al., 2020) for both inversions. No carriers of the inv(12) were found in any of the data sets whereas two inv(10) carriers of European descent were present in gnomAD‐SV.

## 4 DISCUSSION

We used a combination of traditional cytogenetics and molecular approaches to study the features and mechanism of formation for 18 unique large, cytogenetically visible inversions, ranging in size from 8 to 178 Mb. We determined the nt sequence of breakpoint junctions to examine for mutational signatures to potentially infer the likely mechanism that formed the inversion. Mutational signatures have been defined by studying human genomic rearrangements such as *MECP2* duplication syndrome (MIM:300260; Carvalho et al., 2013), Pelizaeus–Merzbacher disease (MIM:312080; Beck et al., 2015), and Potocki–Lupski syndrome (MIM:610883; Beck et al., 2019). Typical signatures that have been observed at the breakpoint junctions are blunt ends, shared nt homology or micromology, presence of templated or random insertions and deletions which may reflect the repair mechanism that leads to its generation (Carvalho et al., 2013; Hastings, Ira, & Lupski, 2009; Weckselblatt & Rudd, 2015; Zhang et al., 2009). In our cohort, 8/13 (62%) inversions with breakpoint junctions determined at nt sequence resolution showed junction features such as blunt ends and very short microhomologies without any additional complexity, which is suggestive of NHEJ/MMEJ repair. However, five inversions (38%) presented templated insertions or copy‐number amplification seemingly mediated by replicative repair involving template switching which is consistent with fork‐stalling and template‐switching (FoSTeS)/MMBIR. Five of the inversions were not detected by either short‐read or linked‐read WGS, possibly due to the presence of large homologous repeats. Though it has previously been proposed that most inversions are mediated through NAHR (Kidd et al., 2008), these results indicate that a fraction of inversions are mediated by mechanisms other than ectopic recombination between inverted repeats.

The incidence of balanced chromosomal aberrations including inversions has been estimated to occur at a rate of 0.522% in an unselected newborn population, of which 15% were pericentric inversions (Jacobs et al., 1992). Only 9.6% of *de novo* inversions are thought to have an associated disease phenotype apparent before the age of 1 year (Warburton, 1991). Disease‐causing recombinant chromosomes as seen in the families with inv(X)(p22.31q28) and inv(3)(p25.3q28), inv(X)(p22.2q26), and inv(X)(p22.3q28) presented here can be generated by meiotic crossing‐over events within an inversion loop. The risk of producing unbalanced gametes from pericentric inversions increases with the size of the inversion, especially when the inverted segments account for greater than 50% of the chromosome size (Morel et al., 2007). Within our own cohort, the inv(X)(p22.31q28) produced unbalanced progeny at least twice over two generations, and the inversion accounted for 93% of the total length of chromosome X. In contrast, the inv(10)(p11.2q21), which seems stable over generations, has an inversion only accounting for 17% of the total size of the chromosome.

Duplication–normal–duplication (DUP–NML–DUP) structures, such as the one detected in inv(X)(p22.31q28) presented here, are relatively rare but are occasionally observed on aCGH analyses. In those cases it is possible that the segment of normal copy number in‐between the duplicated segments is inverted (DUP–INV–DUP; Brand et al., 2015; Gu et al., 2015; Nazaryan‐Petersen et al., 2018). Often, the nested duplications are detected first by chromosomal microarray (CMA) but in the inv(X) case presented here, the inverted segment was large enough to first be identified by cytogenetic analysis and the duplications were later detected by CMA (Xq duplication only visible on high‐resolution aCGH) and WGS. The phenomenon of a large pericentric inversion flanked by duplications was described by Brand et al. (2015) in one proband. The proposed mechanism correlates with what is observed in this case, suggesting that the same mechanism may cause both microscopic SVs, that is, inversions detectable by chromosome analysis, and submicroscopic SVs, that is, only detected by CMA or WGS. Phasing of SNVs within the duplications supports that the duplications were formed concomitantly with the inversion in a one‐step event by MMBIR with iterative template switches (Carvalho & Lupski, 2016).

Five cases in our cohort show mutational signatures suggestive of the replication‐based mechanism MMBIR as generating copy number gains at the junction resulting from template switching (Bahrambeigi et al., 2019; Carvalho & Lupski, 2016; Lee, Carvalho, & Lupski, 2007). In the inv(X)(p22.31q28) (P11758_101), the breakpoints were located within *Alu* elements, but the sequence homology for jct1 (28 bp) and sequence homology for jct2 (32 bp) was not enough for ectopic recombination via NAHR and is more suggestive of MMBIR/FoSTeS as the mechanism of formation (Lee et al., 2007; Song et al., 2018). In addition, two rare SNVs that were not present in dbSNP were identified in one junction, indicative of replicative errors (Beck et al., 2019; Carvalho et al., 2013). In the second complex inversion, inv(12)(p12.2q24.1) (P5371_206), the presence of both a deletion and duplications suggested a replication‐based mechanism of origin; junction analysis revealed short microhomology and a 2 nt insertion. Four out of five breakpoints were located within repeat elements (*Alu*, L1, and simple repeats). The presence of insertions in the breakpoint junctions of three additional inversion cases (mothers of BAB3037, BAB3038, and P4855_144; Table 2), indicate that these inversions may also be formed by MMBIR. In an additional complex inversion (P5513_204), a large deletion spanning 527 bp was detected at the breakpoint junctions. The size of this deletion suggests that end‐processing through resection may have occurred through a repair mechanism such as MMEJ to generate this deletion (Ghezraoui et al., 2014). Therefore, we propose inversions should not be regarded, a priori, as copy‐number neutral without being further investigated as they can present more complex genomic signatures such as DUP–NML–DUP observed by aCGH. The relevance of those findings for the clinical phenotype requires further investigation.

Finally, a total of nine individuals from eight unrelated families from Sweden harbored founder inversions on either chromosome 10 (*n* = 5) or 12 (*n* = 4). Identical breakpoints were observed in all carriers and a common ethnic origin of all individuals suggested that they might have a common ancestor, which was further confirmed by haplotype analysis. The fact that the inv(12)(11.2q13) inversion was not found in population databases but in four affected individuals in this cohort is intriguing. Larger studies of this particular inversion need to be performed to investigate any potential relevance to neurodevelopmental phenotypes or determine if it is indeed a rare normal variant.

In the present study, we used a combination of short‐read WGS, aCGH, and Sanger sequencing and successfully characterized 13 (out of total 18) unique chromosomal inversions to the nt resolution. Among these cases, we found that the most common likely mechanism inferred by the breakpoint junction features, is NHEJ/MMEJ (8/13, 62%) Of note, both seemingly founder inversions (inv(10)(p.13q11.2) and inv(12)(p11.2q13)) showed evidence consistent with identity by descent. The proposed fraction of NAHR‐mediated inversions has been 67% (Kidd et al., 2008), however, in our cohort, the fraction of inversions mediated by mechanisms other than ectopic recombination between inverted repeats were shown to be at least 72% (13/18) with only one‐third representing possible NAHR‐mediated events. When comparing to other chromosomal aberrations like balanced translocations, the underlying mechanism of formation appears to be similar to large chromosomal inversions detailed in this cohort. However, there does appear to be distinct differences in the occurrence of large (greater than 100 bp) CNVs in reciprocal translocations (2%–11%; Nilsson et al., 2017) when compared to our observations of CNVs in large inversions (17%), which suggests replication‐based mechanisms are of greater importance in the latter group.

## 5 CONCLUSIONS

In summary, our study indicates that (i) a proportion of inversion events have hidden complexities and high‐coverage short‐read WGS is a valuable tool to more precisely characterize these inversion events; (ii) NAHR is not the major mechanism underlying the formation of cytogenetically detected chromosomal inversions, instead, the data presented here suggest that at least 72% of chromosomal inversions were mediated by other mechanisms (iii) CNVs and other complexities at the breakpoint may be more prevalent in large unique inversions compared to balanced translocations suggesting a higher incidence of replication‐based mechanisms in the former.

## CONFLICT OF INTERESTS

James R. Lupski has stock ownership in 23andMe, is a paid consultant for Regeneron Pharmaceuticals and is a coinventor on multiple United States and European patents related to molecular diagnostics for inherited neuropathies, eye diseases, and bacterial genomic fingerprinting. The Department of Molecular and Human Genetics at Baylor College of Medicine derives revenue from the chromosomal microarray analysis and clinical exome sequencing offered in the Baylor Genetics Laboratory (https://www.baylorgenetics.com).

## AUTHOR CONTRIBUTIONS

Maria Pettersson and Christopher M. Grochowski performed lab work, analyzed and interpreted data, and wrote the manuscript. Jesper Eisfeldt performed bioinformatics analyses. Josephine Wincent, Amy M. Breman, Sau Wai Cheung, Ana C. V. Krepischi, Carla Rosenberg, James R. Lupski, Jelena Gacic, Jesper Ottosson, Lovisa Lovmar, Elisabeth S. Lundberg, and Daniel Nilsson provided patient samples, clinical information of patients and/or analysis and interpretation of data. Claudia M. B. Carvalho and Anna Lindstrand conceptualized the study, analyzed and interpreted the data, and were major contributors in the writing of the manuscript. All authors have read, edited, and approved the final manuscript.

## ETHICS STATEMENT

The Regional Ethical Review Board in Stockholm, Sweden approved the study (ethics permit number KS 2012/222‐31/3). This ethics permit allows for the use of clinical samples for analysis of scientific importance as part of clinical development. Included subjects were part of clinical cohorts investigated at the respective centers, and the current study reports deidentified results that cannot be traced to a specific individual. For BAB12195 and BAB12196, the informed consents were approved by the Ethics Committee of the Institute of Biosciences, University of São Paulo, Brazil (ethics permit number 2589398). Written informed consent was obtained from the parents of the patient, and family members. All included individuals or legal guardians/parents have given oral consent to be part of these follow‐up clinical investigations.

## Supporting information

Supporting information.

## Data Availability

The consent provided by the research subjects did not permit sharing of the entire genome‐wide data set. BAM files containing all supporting reads for the inversions with WGS data and related variants are deposited in European Nucleotide Archive, project number PRJEB31864.
