# Supplementary material for: Cytogenetically visible inversions are formed by multiple molecular mechanisms
Source: Hum Mutat. 2020 Oct 1;41(11):1979–98. doi: 10.1002/humu.24106 (PMC7702065; doi:10.1002/humu.24106)
Supplement: Supplementary file 1 — Supporting information. [file HUMU-41-1979-s001.pdf]

Calculating the haplotype index

The haplotype index (HI) is a measurement of the similarity of the haplotypes of two individuals (A and B), across a region R. The haplotype index is similar to the jaccard index, and is calculated using the following formula:

1: For each individual, extract all Homozygous SNVs within region R, such that:

$$R_{\mathrm{hom}A} = \{All\ homozygous\ SNVs\ of\ A\ within\ R\}$$

$$R_{\mathrm{hom}B} = \{All\ homozygous\ SNVs\ of\ B\ within\ R\}$$

2. For each individual, extract all SNVs within region R such that:

$$R_A = \{All\ SNVs\ of\ A\ within\ R\}$$

$$R_B = \{All\ SNVs\ of\ B\ within\ R\}$$

3. Calculate the HI:

$$HI_A = \frac{|R_A \cap (R_{\mathrm{hom}A} \cup R_{\mathrm{hom}B})|}{|R_{\mathrm{hom}A} \cup R_{\mathrm{hom}B}|}$$

$$HI_B = \frac{|R_B \cap (R_{\mathrm{hom}A} \cup R_{\mathrm{hom}B})|}{|R_{\mathrm{hom}A} \cup R_{\mathrm{hom}B}|}$$

Note HI_A may be different from HI_B.

If the two individuals share a haplotype within region R, all variants within $(R_{\mathrm{hom}A} \cup R_{\mathrm{hom}B})$ must be present in both individuals, either as homozygous or heterozygous variants. Hence, if the individuals share a haplotype, the intersect $R_A \cap (R_{\mathrm{hom}A} \cup R_{\mathrm{hom}B})$ will be large.

Fig. S1. Classical karyotyping was first used to discover a large genomic inversion was present. Short-read whole genome sequencing and/or array comparative genomic hybridization was used to refine the precise location of the inversion. If no junction was identified by short-read WGS we attempted linked-read WGS. If the junction contained a CNV and could be inferred from the high resolution aCGH or short-read WGS, primers were designed to Sanger-validate the proposed junctions. If there were further complexities, including junction-level copy number variation, we implemented droplet digital PCR to confirm the structure. Link-read WGS was also used for subsequent phasing studies unrelated to architecture resolution.

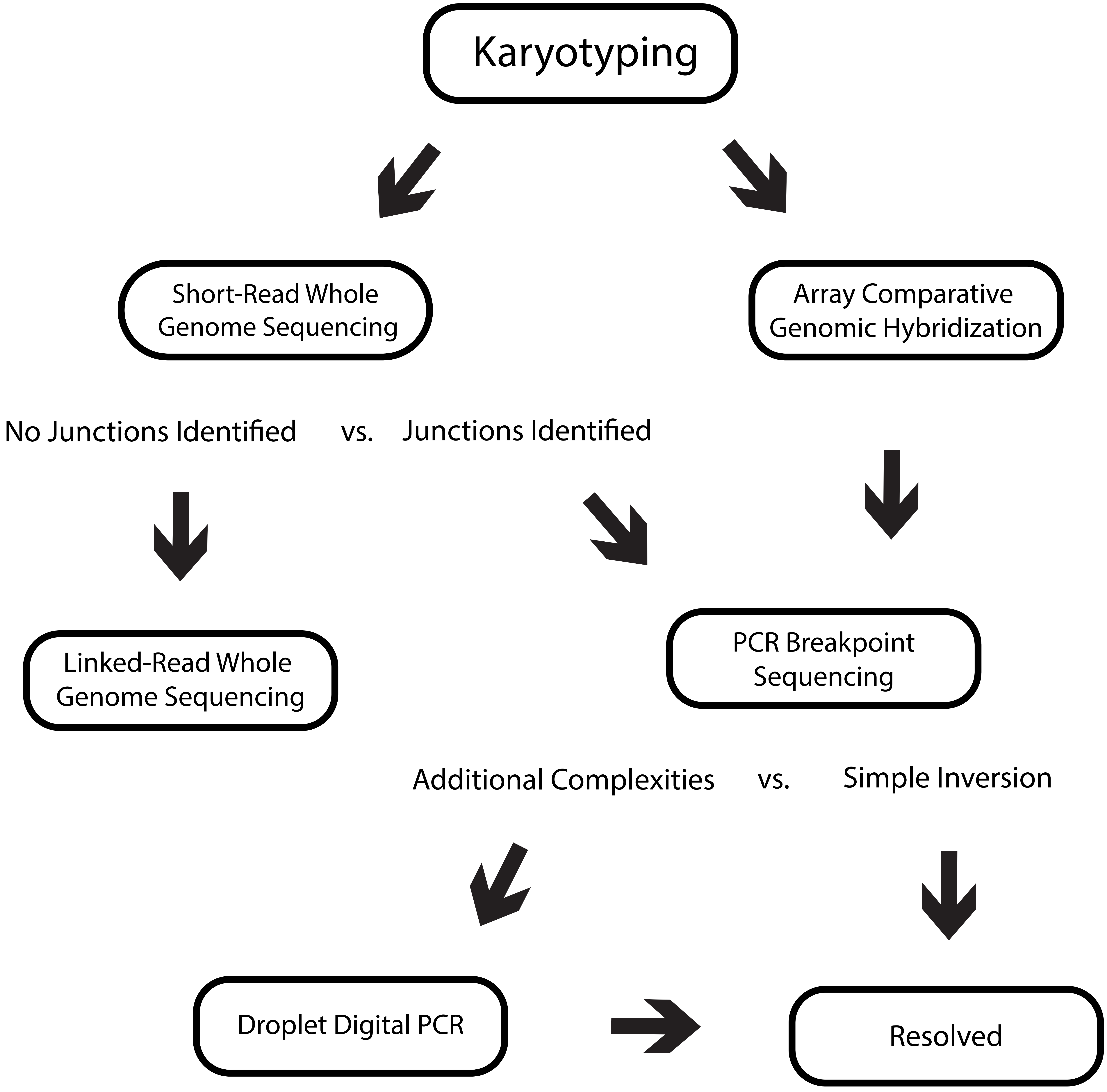

Fig. S2. Purple: microhomology, pink: insertion, underlined pink: imperfect templated insertion copied from adjacent p-arm region (underlined blue), red: single nucleotide variant, lower case bold letters: deletion

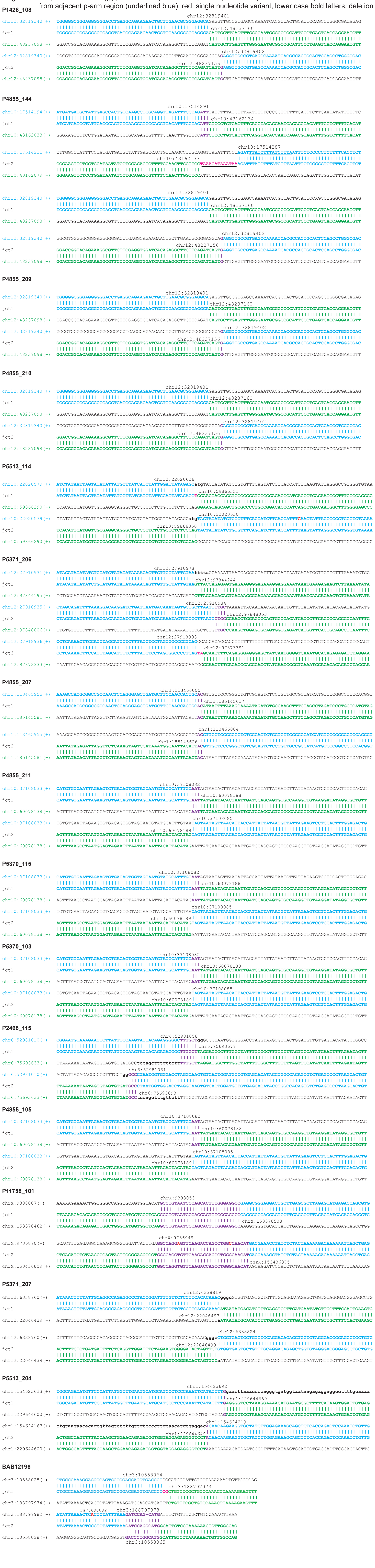

Fig. S3. Breakpoint PCR confirmed that only junction (jct) 2 was present in the carriers of the derivative and inverted chromosome X (A) . Breakpoint junctions 1 and 2 alignment revealed two single nucleotide variants in junction 1,none of them were present in the dbSNP database (B).

A

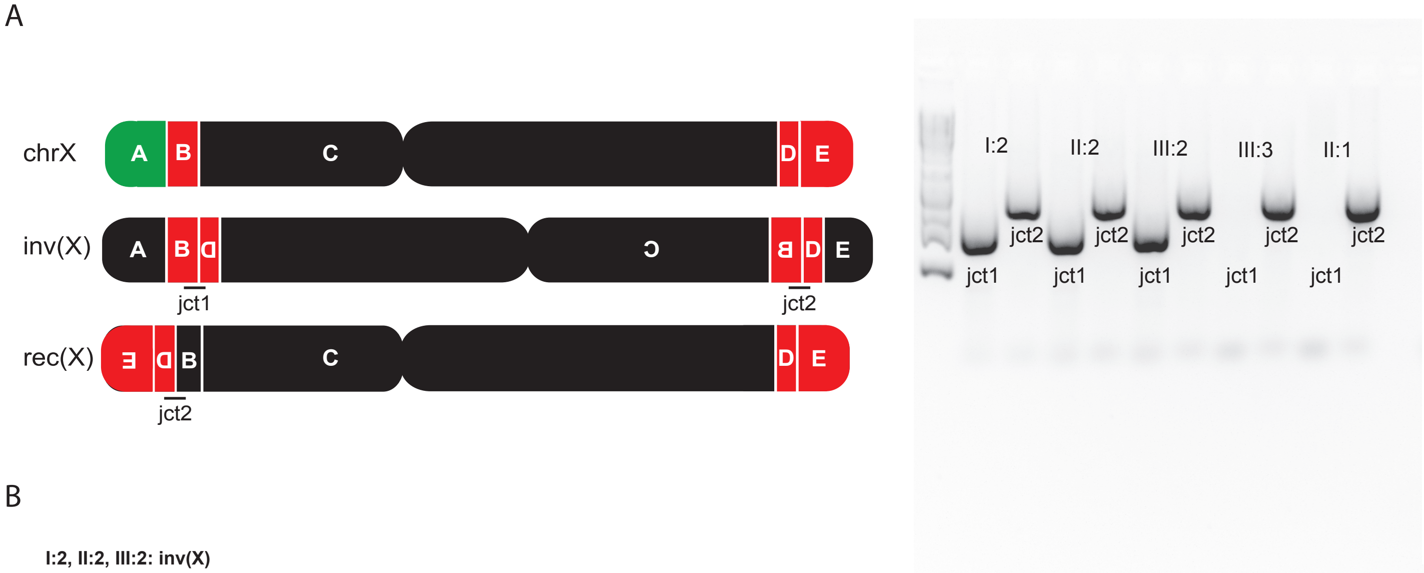

B

I:2, II:2, III:2: inv(X)

```
chrX:9736887(+)    CTTTGAGAGGCCAAAGCGGGTGGATCACTTGAGGCCAGGAGTTCAAGACCAGCCTGGCCAACATGACGAAACCTATCTCTACTAAAAAGACAAAAAATTAGCTGAGTGT
jct1               ACATCTGTAACCCCAGTACTTGGGGAGGCCGTGGCCAGGTGTTCAAGACCAGCCTGGGCAACATGACGAAACCTATCTCTACTAAAAAGACAAAAAATTAGCTGAGTGT
chrX:153436828(-)  ACATCTGTAACCCCAGTACTTGGGGAGGCCGTGGCCAGGTGTTCAAGACCAGCCTGGGCAACATAGCAAGATCCCATCTCTACAAATAATAATAATTTTTAAAAAGTTA

chrX:9388003(-)    GCTGGTCTCATACTCTAAGCGCTCAAGCAGTCCTCCCGCCTCGGCCTCCCAAAGTGCTGGGATTACAGGCATGTGCCACTGCACCTGGCCCACCAGTTTTCTTTTTGCA
jct2               GCTGGTCTCATACTCTAAGCGCTCAAGCAGTCCTCCCGCCTCGGCCTCCCAAAGTGCTGGGATTACAGGCCTGAGCCACCATGCCCAGCCAATCTCTGTCTTTTAATTG
chrX:153378456(+)  GGCTGCTCTTGAACTCCTGACCTCAGGTGATGCACCCACCTTGGCCTCCCAAAGTGCTGGGATTACAGGCCTGAGCCACCATGCCCAGCCAATCTCTGTCTTTTAATTG
```

II:1, III:3: rec(X)

```
chrX:9388003(-)    GCTGGTCTCATACTCTAAGCGCTCAAGCAGTCCTCCCGCCTCGGCCTCCCAAAGTGCTGGGATTACAGGCATGTGCCACTGCACCTGGCCCACCAGTTTTCTTTTTGCA
jct2               GCTGGTCTCATACTCTAAGCGCTCAAGCAGTCCTCCCGCCTCGGCCTCCCAAAGTGCTGGGATTACAGGCCTGAGCCACCATGCCCAGCCAATCTCTGTCTTTTAATTG
chrX:153378456(+)  GGCTGCTCTTGAACTCCTGACCTCAGGTGATGCACCCACCTTGGCCTCCCAAAGTGCTGGGATTACAGGCCTGAGCCACCATGCCCAGCCAATCTCTGTCTTTTAATTG
```

Fig. S4. Phenotypic presentation for each identified member of the family. Droplet digital PCR targeting junction 2 shows similar levels of amplification for all family members containing the junction.

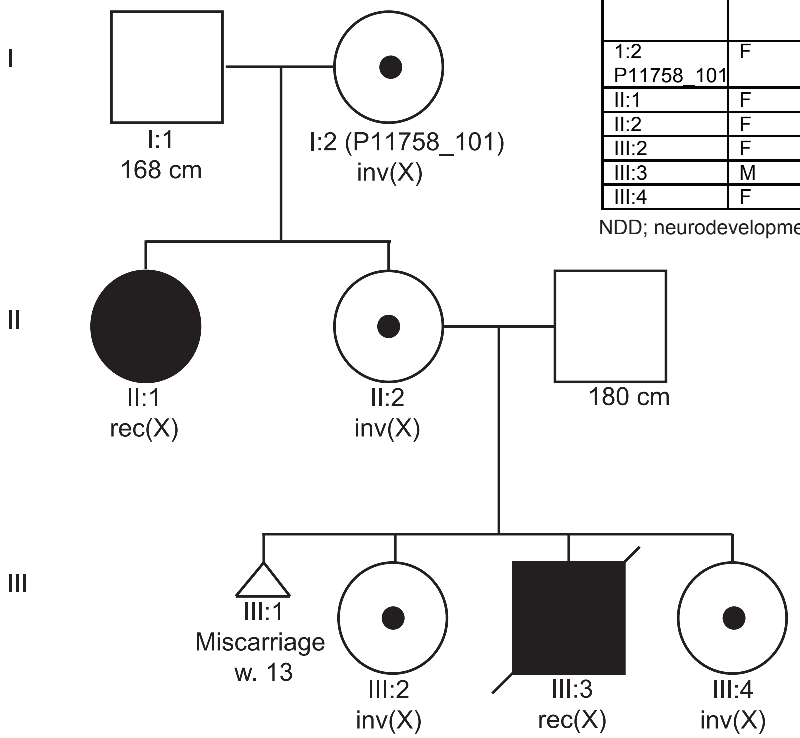

ID	Gender	Short stature	Madelung deformity	Short forarms /shanks	Skeletal /joint pains	Congenital malformations	NDD
1:2 P11758_101	F	No	No	No	No	No	No
II:1	F	Yes	Yes	Yes	Yes	No	Yes
II:2	F	Yes	No	No	No	No	No
III:2	F	Yes	No	Yes	Yes	No	No
III:3	M	N.i.	N.i.	N.i.	N.i.	Yes	N.i.
III:4	F	N.i.	N.i.	N.i.	N.i.	No	N.i.

NDD; neurodevelopmental disorder, N.i.; no information

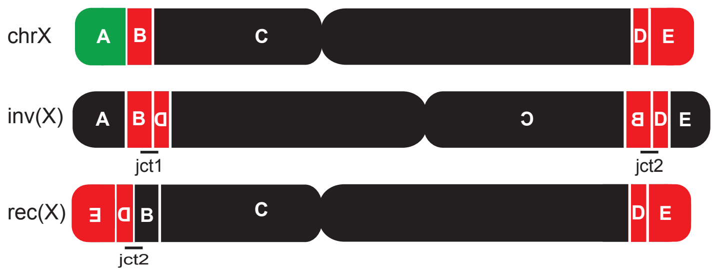

Digital droplet PCR - Junction 2 (present in both inversion and recombinant chromosome carriers)

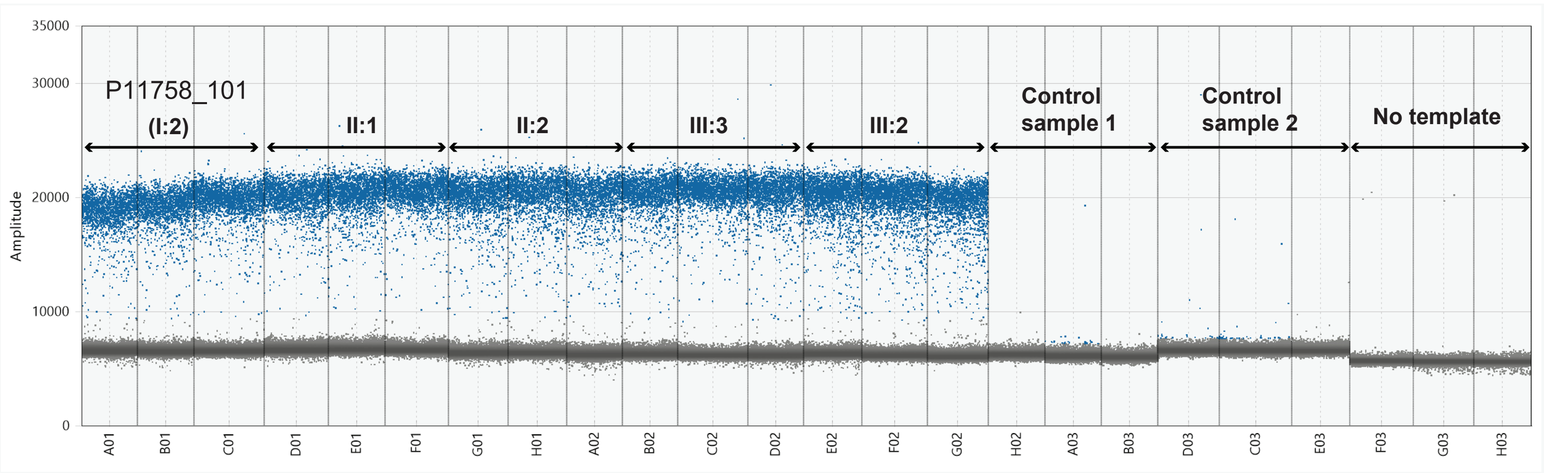

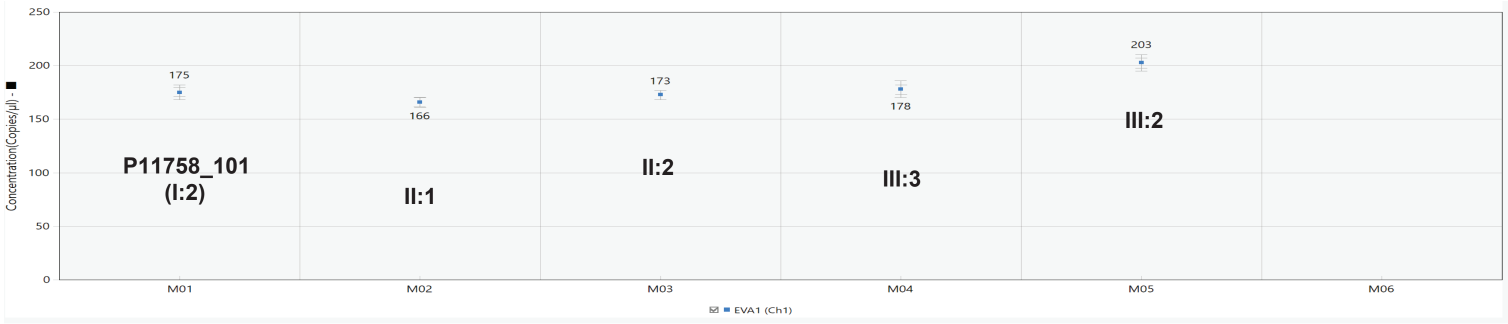

Fig. S5. Analysis of molecules bridging both duplications by linked-read sequencing showed that they were present on the same allele in cis.

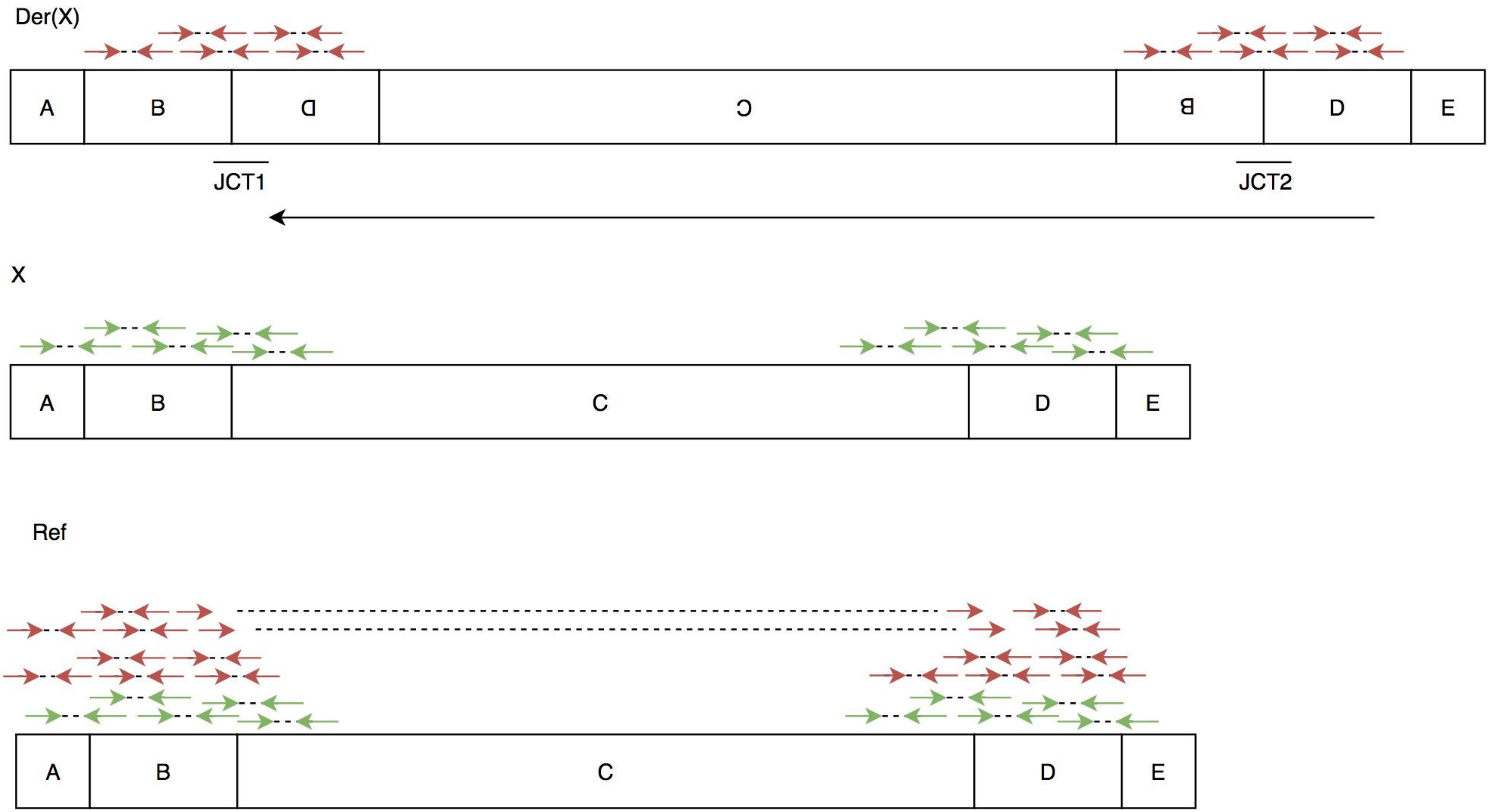

Fig. S6. Purple - microhomology, pink - insertion (arrows show direction of templated insertion copied from adjacent segments), bold lower case letters - deletion.

Chiang_2012_BSID23

```
chr5:24272247(+)   CCCTTTGTAAATTACCTCAGTAGTCTTTGATAACTTCCTTGTTTTCTACTCTGACAAGATATTTATAGAGATCTAACCAATCTGGGACATTTTAATACTGTAATTTGTGT
junction 1         CCCTTTGTAAATTACCTCAGTAGTCTTTGATAACTTCCTTGTTTTCTACTCTGACATCGTGTCAGGCAGAGGTATAACCTTGTTATTGTTTTCATTTGAAGATTAATTAT
chr5:88400789(-)   TATTCTCTTCTGGGGAAAAAGTTAGTGCATTTTTGGTTGTATGCTGGATAGTCGTATCGTGTCAGGCAGAGGTATAACCTTGTTATTGTTTTCATTTGAAGATTAATTAT

chr5:24272247(+)   CCCTTTGTAAATTACCTCAGTAGTCTTTGATAACTTCCTTGTTTTCTACTCTGACAAGATATTTATAGAGATCTAACCAATCTGGGACATTTTAATACTGTAATTTGTGT
junction 2         AAAAAGTTAGTGCATTTTTGGTTGTATGCTGGATAGCTTGTCATATAAATATGACAAGATATTTATAGAGATCTAACCAATCTGGGACATTTTAATACTGTAACTTGTGT
chr5:88400774(-)   AAAAAGTTAGTGCATTTTTGGTTGTATGCTGGATAGtcgtATCGTGTCAGGCAGAGGTATAACCTTGTTATTGTTTTCATTTGAAGATTAATTATGATATAAGGAGATGT
```

Chiang_2012_BSID27

```
chr3:189669134(+)   GGGTGATTGGATGCTTGTTGCTTTATGTCCTATTTGACCCTTTGGTTTTAAATGAGGTAAATTGCTCCAATTAACTCCTGTTGGAGATGGTTTTCTTTCAATGATTCCTT
junction 1          GGGTGATTGGATGCTTGTTGCTTTATGTCCTATTTGACCCTTTGGTCTCTACCAAGGAGACTGACCTGTTCTTTCCTCTGTGATTCCATGGCAGTGAGTTGATATTTGAT
chr3:111406101(-)   TGCCTTTCCATTTTAATTACCATCATTAGTCTGTGAGCTCACCTGACTCTACCAAGGAGACTGACCTGTTCTTTCCTCTGTGATTCCATGGCAGTGAGTTGATATTTGAT

chr3:189669134(+)   GGGTGATTGGATGCTTGTTGCTTTATGTCCTATTTGACCCTTTGGTtttaaatgaGGTAAATTGCTCCAATTAACTCCTGTTGGAGATGGTTTTCTTTCAATGATTCCTT
junction 2          TTGTCCCTTGCCTTTCCATTTTAATTACCATCATTAGTCTGTGAGCTCACCTGAGGGTAAATTGCTCCAATTAACTCCTGTTGGAGATGGTTTTCTTTCAATGATTCCTT
chr3:111406114(-)   TTGTCCCTTGCCTTTCCATTTTAATTACCATCATTAGTCTGTGAGCTCACCTGACTCTACCAAGGAGACTGACCTGTTCTTTCCTCTGTGATTCCATGGCAGTGATCCTT
```

Chiang_2012_BSID33

```
                                                   rs2114738
chr2:171827193(+)   GAGTTTGCAGAGCCCTGTGTTATCCTGGTAGTAACACAGCTGAGTCTGGGGAGAGGGGAGGGATAGCTGGGAAACTGAGGTTTCCTTACAGTCAGCGGGGCCAGAACCCG
junction 1          GAGTTTGCAGAGCCCTGTGTTATCCTGGCAGTAACACAGCTGAGTCTGGGGTACACATCTGTGGTCCCATCTACTTGGGGGACTGAGGAGAGAGGTCACTTGAGCTGAAG
chr2:32310389(-)    ggtgagattttatctctaccaaaaaatttaaaaattaaccatgattggggaTACACATCTGTGGTCCCATCTACTTGGGGGACTGAGGAGAGAGGTCACTTGAGCTGAAG

chr2:171827181(+)   GAGTTTGCAGAGCCCTGTGTTATCCTGGTAGTAACACAGCTGAGTCTGGGGAGAGGGGAGGGATAGCTGGGAAACTGAGGTTTCCTTACAGTCAGCGGGGCCAGAACCCG
junction 2          GTACTACCTCCCCGATTCTTCTGTTTCTTCACCCTGAAAATAACTAACCAGAGAGGGGAGGGATAGCTGGGAAACTGAGGTTTCCTTACAGTCAGCGGGGCCAGAACCCG
chr2:32310663(-)    GTACTACCTCCCCGATTCTTCTGTTTCTTCACCCTGAAAATAACTAACCAtattcatccccttaaacaggtcaatttcttatgttatagaattatcagtattatatacta
```

Chiang_2012_BSID35

```
chr7:157577796(+)   TCCAGAATGTTGGCACAGCGGCGAGAAGGCTGACCGAACACCGAGAAGCAGCCAGCACAGGCTCTCCACAGGGGCTCTGGCATGGAGCAGGATAACCCGGGCCCACGTGG
junction 1          TCCAGAATGTTGGCACAGCGGCGAGAAGGCTGACCGAACACCGAGAACAGCCTGGGTGACAGAGCGAGACCAGAATTCTAAAACAAAAGAGGATAGGATAGATATACAAT
chr7:69685924(-)    CAGGAGGCAGAGCCCAGCAGTTGCAGGCTGTAGTGACCactgcactcCAGCCTGGGTGACAGAGCGAGACCAGAATTCTAAAACAAAAGAGGATAGGATAGATATACAAT

chr7:157577796(+)   AATGTTGGCACAGCGGCGAGAAGGCTGACCGAACACCGAGAAGCAGCCAGCACAGGCTCTCCACAGGGGCTCTGGCATGGAGCAGGATAACCCGGGCCCACGTGGGGACA
junction 2          CAGGAGGCAGAGCCCAGCAGTTGCAGGCTGTAGTGACCTGTAGCAGCCAGCACAGGCTCTCCACAGGGGCTCTGGCATGGAGCAGGATAACCCGGGCCCACGTGGGGACA
chr7:69685924(-)    CAGGAGGCAGAGCCCAGCAGTTGCAGGCTGTAGTGACCACTGCACTCCAGCCTGGGTGACAGAGCGAGACCAGAATTCTAAAACAAAAGAGGATAGGATAGATATACAAT
```

Chiang_2012_BSID37

```
chr12:82318978(+)   GTCCCACAATTCTTAGATATTCTATTTCAAATTTTAATCTAATTTTCTTTTTGATTTTCAGTTTGGGAAGTTTCTATAGACTTATATTCAAGCTCACCAGTTATTTTCTT
junction 1          GTCCCACAATTCTTAGATATTCTATTTCAAATTTTAATCTAATTTTCTTTTAATAATTTTTCTTGTTACAAAAGCAATGCATGAACATTTGAGAAAAACTAGAAGACAAG
chr12:13955767(-)   GGACCCTGCACAGTTCAAACCTGTGTGTTCCCGAGTTAACTGTATCACTTTAATAATTTTTCTTGTTACAAAAGCAATGCATGAACATTTGAGAAAAACTAGAAGACAAG

chr12:82318978(+)   GTCCCACAATTCTTAGATATTCTATTTCAAATTTTAATCTAATTTTCTTTTTGATTTTCAGTTTGGGAAGTTTCTATAGACTTATATTCAAGCTCACCAGTTATTTTCTT
junction 2          GGACCCTGCACAGTTCAAACCTGTGTGTTCCCGAGTTAACTGTATCACTTTTGATTTTCAGTTTGGGAAGTTTCTATAGACTTATATTCAAGCTCACCAGTTATTTTCTT
chr12:13955767(-)   GGACCCTGCACAGTTCAAACCTGTGTGTTCCCGAGTTAACTGTATCACTTTAATAATTTTTCTTGTTACAAAAGCAATGCATGAACATTTGAGAAAAACTAGAAGACAAG
```

Chiang_2012_BSID47

```
chr3:114735909(+)   TATTCAAATAGGGAGAGAGGAAGTTAAATTATCTTTGTTTGCAGATAACAGGATCCTATGTCTAGAAAAGCACATCATCTCAGCCCAAAACCTTCTTAAGCTGATAAGCA
junction 1          TATTCAAATAGGGAGAGAGGAAGTTAAATTATCTTTGTTTGCAGATAACAATGATTCCTGAAGAAATCTTCATACTTTTGAGATGGGAAATCTTTTCTCTAACTTAGAGG
chr3:80706827(-)    CGGAAGAAATCTTATTCTTCAAATTGTCCTTGCAGGTTCCTCCTGCAAGGATGATTCCTGAAGAAATCTTCATACTTTTGAGATGGGAAATCTTTTCTCTAACTTAGAGG

chr3:114735909(+)   TATTCAAATAGGGAGAGAGGAAGTTAAATTATCTTTGTTTGCAGATAACAGGATCCTATGTCTAGAAAAGCACATCATCTCAGCCCAAAACCTTCTTAAGCTGATAAGCA
junction 2          GAAGAAATCTTATTCTTCAAATTGTCCTTGCAGGTTCCTCCTGCAAGGATGGATCCTATGTCTAGAAAAGCACATCATCTCAGCCCAAAACCTTCTTAAGCTGATAAGCA
chr3:80706827(-)    GAAGAAATCTTATTCTTCAAATTGTCCTTGCAGGTTCCTCCTGCAAGGATGATTCCTGAAGAAATCTTCATACTTTTGAGATGGGAAATCTTTTCTCTAACTTAGAGGCA
```

Watson_2016

```
chr7:93599475(+)   ATGAAAGAGGGAAATAAGTGGATTTTGCAAAGAATATTCACCAAGACCTTTGTTTAATAGAGGGGAGAAAAAGAAATAATTAAATACTAGCAGAAAACAGGGTTGCCTCT
junction 1         ATGAAAGAGGGAAATAAGTGGATTTTGCAAAGAATATTCACCAAGACCTTTGTTTAATTTAAAATAGTCTTCTGCAACTTGATTTTGTTCACCCAAGGGTATGTTTTCAA
chr7:27762369(-)   TATTTAAACTTTGCACTGTTTCACCTTCCTTGTTTTGTGCTTTATAAAAACAACATATTTAAAATAGTCTTCTGCAACTTGATTTTGTTCACCCAAGGGTATGTTTTCAA

chr7:93599475(-)   CAAAGAGGCAACCCTGTTTTCTGCTAGTATTTAATTATTTCTTTTTCTCCCCTCTATTAAACAAAGGTCTTGGTGAATATTCTTTGCAAAATCCACTTATTTCCCTCTTT
junction 2         ATCTTGAAAACATACCCTTGGGTGAACAAAATCAAGTTGCAGAAGACTATTTTAAATTAAACAAAGGTCTTGGTGAATATTCTTTGCAAAATCCACTTATTTCCCTCTTT
chr7:27762369(+)   ATCTTGAAAACATACCCTTGGGTGAACAAAATCAAGTTGCAGAAGACTATTTTAAATATGTTGTTTTTATAAAGCACAAAACAAGGAAGGTGAAACAGTGCAAAGTTTAA
```

Fig. S7.

Breakpoint junction sequences were confirmed to be identical to previously published cases, supporting that the inv(10)(p11.2q21) is identical by descent.

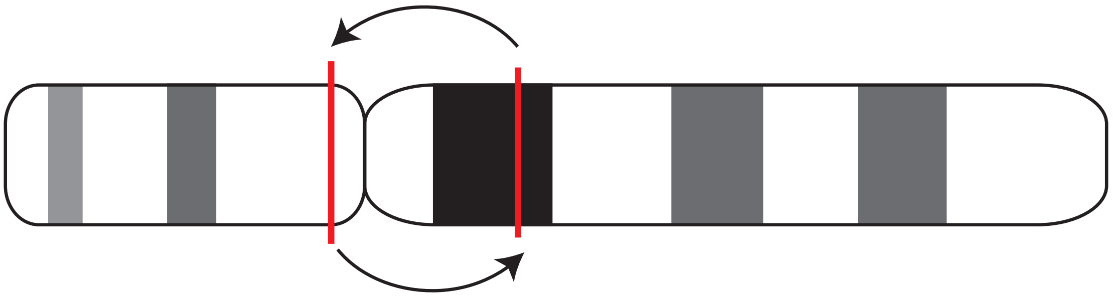

```
chr10p_intergenic (+)   GTAGTAATGTATGCATTTGTAATAGTAATAGTTAACATTACCA
                        |||||||||||||||||||||||
junction 1              GTAGTAATGTATGCATTTGTAATTATGAATACACTAATTGATC
                                            |||||||||||||||||||||||
chr10q_intergenic (-)   ATAATAATTACATTACATAGAATTATGAATACACTAATTGATC

chr10p_intergenic (+)   AGTAATGTATGCATTTGTAATAGTAATAGTTAACATTACCATT
                                            |||||||||||||||||||||||
junction 2              ATAATAATTACATTACATAGTAGTAATAGTTAACATTACCATT
                        ||||||||||||||||||||
chr10q_intergenic (-)   ATAATAATTACATTACATAGAATTATGAATACACTAATTGATT
```

Table S1. Primers for breakpoint PCRs.

Classic inversions

Inversion	Forward primer Junction 1 (5´->3´)	Reverse primer Junction 1 (5´->3´)	Forward primer Junction 2 (5->3´)	Reverse primer Junction 2 (5->3´)
inv(1)(p13q25)	acgcagtgataggagacgtataaat	acacagcaggggattaatatagctt	attgctcttgcagaggttacagat	agaaatatcttaccattgcaatcca
inv(3)(p25.3q28)	tgggtgttcagggatgtcta	agagaccatggcttctatatcc	ggaagagaagcaggacagatg	ctggcccacgtgtaacttat
inv(3)(p25.3q28) Sequencing primers	cttggctttgattgacactctg	ctgagcctggtttcttcatcag	ggcgagagccctgtaaatatc	taggtttggctccaggaaag
inv(6)(p12.1q13)	tatatacacctccaactctgactatgtctt	ttttcttcatcaaagtatctaacaaaacat	ttttctagctagtggttcacatttactaag	cttatgtaaacctcttgtctcagtgttact
inv(10)(p11.2q21)	gttcaaaagaacaaatgatctagcc	ggaaattctccaaattaacttccat	ttcacagtatttttctgcctatgct	ttagcaaagaaagatcaccgtattc
inv(10)(p12q21)	gtctcggtgcttaccatgattatac	ggactataaggtcaaaacctccagt	tttgtcagggtaccattcataaaat	ctcacagatcaccaacaacctgtat
inv(10)(p13q11.2)	cttgaatttgaaaatggcagatctat	agacaaacaaaaagacaagagtaacctc	aacaagggtatagtttgtagtttggatatt	agtttcttagtcctgagttctagtttgatt
inv(12)(p11.2q13)	agagatgcaaggaactcctaaatcag	acattacaaagacaaatgcatgttatagat	aacaggaatcattgttcttatttgtattt	ctctcctgcctactcacgataaataat
inv(12)(p12.2p13.3)	atgaggtgcctggtgattttc	gattgtttacaaagcattctgaggt	tgctgttgataaatgacaaagtgtt	cataaggtatgcactgaagtgtctg
inv(1)(q21.3q42.13	cccccacctgttagttcttttt	tagcctcttttcacactggtagttc	agaacaagagcaaaactctgtctca	actagacacaggtaaggaaccctct

Complex inversions

Inversion	Forward primer Junction 1 (5´->3´)	Reverse primer Junction 1 (5´->3´)	Forward primer Junction 2 (5->3´)	Reverse primer Junction 2 (5->3´)	Forward primer Junction 3 (5´->3´)	Reverse primer Junction 3 (5->3´)
inv(12)(p11.2q24.1)	ggagagtaatgtgtcctgactccta	acagtgtgtagtgatgaaatcagga	ggcagcttcacatatttttgtattt	ccactgtacctgcctaattctttta	ttttagttaagctctttgagggtca	gttgcctatgtgtttaagcaacttc
inv(X)(p22.31q28)	tagaggaagacgacacatctcaaat	ctgagtctaattgttgctgtgatagagac	tcctaagcagacgtaaagggattat	ttcctgattgatctaacagctaaca	-	-

Table S2. Molecular karyotypes of all successfully sequenced cases.

Sample ID	Molecular karyotype
P1426_108, P4855_209, P4855_210	46,XY,inv(12)(p11.2q13).seq[GRCh37] inv(12)(pter→p11.21::q13.11→p11.21::q13.11→qter) chr12:g.[32819401_cen_48237160inv]
P4855_144	46,XX,inv(10)(p13q11.2).seq[GRCh37] inv(10)(pter→p12.33::q11.21→p12.33::q11.21→qter) chr10:g.[17514291_cen_43162134inv::TAAAGATAAATAA]
	46,XX,inv(12)(p11.2q13).seq[GRCh37] inv(12)(pter→p11.21::q13.11→p11.21::q13.11→qter) chr12:g.[32819401_cen_48237160inv]
P4855_105, P4855_211, P5370_115, P5370_103, P5370_113	46,XY,inv(10)(p11.2q21).seq[GRCh37] inv(10)(pter→p11.21::q21.1→p11.21::q21.1→qter) chr10:g.[37108082_cen_60078188inv]
P2468_115	46,XX,inv(6)(p11q13).seq[GRCh37] inv(6)(pter→p12.1::q13→p12.1::q13→qter) chr6:g.[75693678_75693692del;52981058_cen_75693677inv]
P11758_101	46,X,inv(X)(p22.31q28).seq[GRCh37] inv(X)(pter→p22.31::q28→p.22.31::q28→qter) chrX:g.[9388053_9736949dup;9736950_cen_153378508inv;153378509_153436856dup]
P5513_114	46,XY,inv(10)(p12q21).seq[GRCh37] inv(10)(pter→p12.31::q21.1→p.12.31::q21.1→qter) chr10:g.[59866352_59866354del;22020626_cen_59866350inv::T]
P5371_206	46,XX,inv(12)(p11.2q24.1).seq[GRCh37] inv(12)(pter→p11.22::q23.1→p11.22::q23.1→qter) chr12:g.[27910979_27910983del; 27910978_27918930_cen_97844244_97848048_97873453inv::TA]
BAB12196	46,XX,inv(3)(p25.3q28).seq[GRCh37] inv(3)(pter→p25.3::q28→p25.3::q28→qter) chr3:g.[10558064_cen_188797973inv]

P5513_204	46,XX,inv(1)(q21.3q42.13).seq[GRCh37]<br>chr1:g.[154623693_154624218del;154623692_229644659inv]
P5371_207	46,XX,inv(12)(p12.2p13.3).seq[GRCh37)<br>chr12:g.[6338820_6338823del;6338819_22046497inv]

Table S3. Breakpoint junction characteristics of 25 unique junctions reported here and 14 reanalyzed previously published junctions.

	All Junctions		Our cohort		Reanalysis cohort	
**Total number**	39	100%	25	100%	14	100%
**Microhomology, total**	18	46%	12	48%	6	43%
**<2 nt**	5	13%	3	12%	2	14%
**2-20 nt**	11	28%	7	28%	4	29%
**>20 nt**	2	5%	2	8%	0	0%
**Insertions, total**	8	21%	5	20%	3	21%
**<2 nt**	2	5%	1	4%	1	7%
**2-20 nt**	6	15%	4	16%	2	14%
**Templated insertions**	4	10%	3	12%	1	7%
**Base deletions, total**	12	31%	8	32%	4	29%
**<2 nt**	1	3%	1	4%	0	0%
**2-20 nt**	9	23%	6	24%	3	21%
**>20 nt**	2	5%	1	4%	1	7%

nt; nucleotide

Previously Published Cohorts:

Chiang C, Jacobsen JC, Ernst C, Hanscom C, Heilbut A, Blumenthal I, Mills RE, Kirby A, Lindgren AM, Rudiger SR and others. 2012. Complex reorganization and predominant non-homologous repair following chromosomal breakage in karyotypically balanced germline rearrangements and transgenic integration*. Nature Genetics 44*(4):390-7, S1.

Watson CM, Crinnion LA, Harrison SM, Lascelles C, Antanaviciute A, Carr IM, Bonthron DT, Sheridan E. 2016. A Chromosome 7 Pericentric Inversion Defined at Single-Nucleotide Resolution Using Diagnostic Whole Genome Sequencing in a Patient with Hand-Foot-Genital Syndrome. *PLoS One 11*(6):e0157075.
